# Amplification-free detection of plant pathogens by improved CRISPR-Cas12a systems: a case study on phytoplasma

**DOI:** 10.3389/fpls.2025.1544513

**Published:** 2025-03-06

**Authors:** Joseph R. Lagner, Eric A. Newberry, Yazmín Rivera, Liyang Zhang, Christopher A. Vakulskas, Yiping Qi

**Affiliations:** 1 Department of Plant Science and Landscape Architecture, University of Maryland, College, Park, MD, United States; 2 Animal Plant Health Inspection Service, Plant Protection and Quarantine, Science and Technology, Plant Pathogen Confirmatory Diagnostics Laboratory, United States Department of Agriculture, Laurel, MD, United States; 3 Integrated DNA Technologies, Coralville, IA, United States; 4 Institute for Bioscience and Biotechnology Research, University of Maryland, Rockville, MD, United States

**Keywords:** CRISPR-Cas12a, pathogen detection, phytoplasma, fluorescence based detection, lateral flow assay (LFA), LbCas12a-ultra, stem-loop probe

## Abstract

CRISPR-based disease detection has the potential to profoundly change how pathogens are detected in plant materials. However, there has been a lack of research directed into improving explicitly the CRISPR components that define these detection assays. To fill this technology gap, we have designed and optimized our CRISPR-Cas12a based detection platform by showcasing its capability of detecting a plant pathogen group of rising importance, *Candidatus* Phytoplasma. Most assays utilize isothermal pre-amplification steps, which may boost sensitivity yet often lead to false positives. Aiming for a pre-amplification-free assay to maintain accuracy, we screened multiple Cas12a orthologs and variants and found LbCas12a-Ultra to be the most sensitive Cas12a. We further improved the detection system by using stem-loop reporters of various sizes and found 7nt stem-loop significantly outperformed other stem-loop sizes as well as the commonly used linear reporters. When the 7nt stem-loop reporter was combined with the best-performing LbCas12a-Ultra, we found a 10-fold increase in sensitivity over the standard LbCas12a with the linear reporter detection assay. To enhance the coverage of highly diverse phytoplasmas, we tested a multiplex detection method predicted to target nearly 100% of all documented phytoplasma species on NCBI. A lateral flow assay was also developed to accommodate instrument-free detection with the optimized reagents. Our study demonstrates an improved CRISPR-Cas12a detection system that has wide applications for plant pathogen detection and can be easily integrated into almost any other Cas12a-based detection platform for boosted sensitivity.

## Introduction

Disease detection has received massive progress when the need for rapid, specific, and sensitive test assays for pathogens arose during the COVID-19 pandemic (Javalkote et al., 2020; Li et al., 2021; López-Valls et al., 2022; Nouri et al., 2021; Patchsung et al., 2020; Sun et al., 2021; Yang et al., 2023). However, despite the huge attention COVID-19 received, there are still many other impactful, non-human diseases circulating the globe that would benefit from having a sensitive detection assay. Plant diseases for instance, could create huge global problems by devasting delicate ecosystems, decreasing biodiversity, and minimizing a source of food for many species (He and Creasey Krainer, 2020). Researchers have established CRISPR-based assays for several harmful plant pathogens (Bhat et al., 2022; Sánchez et al., 2022; Marqués et al., 2022). These assays demonstrate the capacity of sensitive, specific, and even potential on-site testing possible with CRISPR-based detection. These assays utilize a unique behavior known as the trans-cleavage characteristics of type V CRISPR-Cas proteins, with Cas12a being the most common for detection. Type V Cas proteins are characterized by having a distinct ability to indiscriminately cleave single-stranded genetic material after successfully identifying a complimentary match to the crRNA (Chen et al., 2018; Swarts and Jinek, 2019; Tong et al., 2021; Yan et al., 2019).

**Figure 1:**
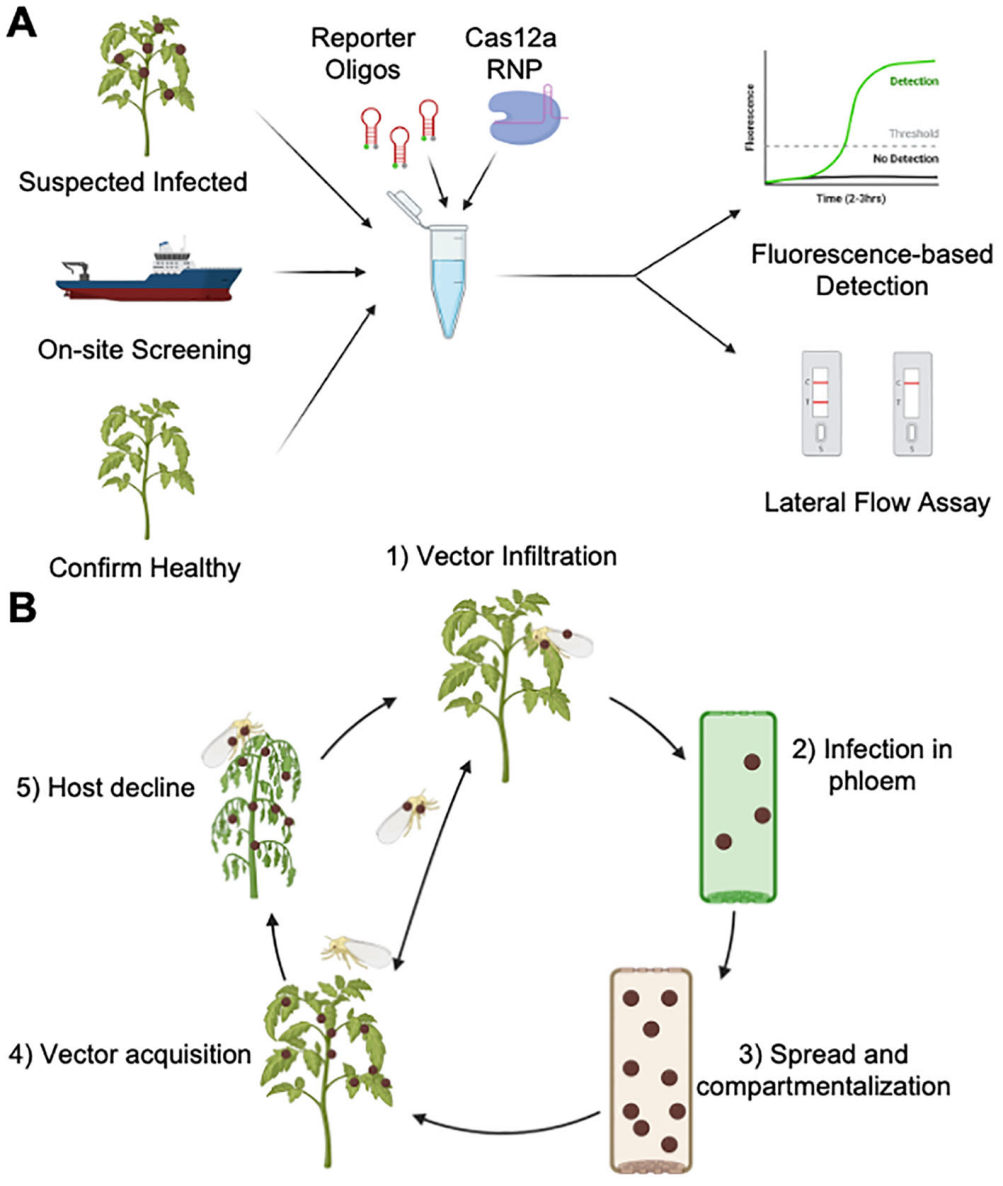
CRISPR-Cas12a based detection of plant pathogens. **(A)** Screening for plant pathogens using CRISPR technology. The schematic depicts how a CRISPR-based detection platform could be best utilized in monitoring of plant pathogens in a laboratory using fluorescence-based assays, or on-site using Lateral Flow Assays (LFA). **(B)** A plant disease cycle with an example of phytoplasmas. The disease is commonly vectored by ‘piercing-sucking’ insects mostly in the Hemiptera family. The most effective strategy is removal or eradication of infected plants before the insects feed and acquire phytoplasmas in their salivary glands. Hence, there is a strong need for a sensitive test that can detect the presence of phytoplasmas in plants that are heavily infested but still appear to be healthy. Created with BioRender.com.

In a typical CRISPR-based detection assay, short DNA or RNA reporter oligonucleotides are added into the system with a fluorophore attached on one end and a quencher attached to the other end (**Figure 1A**). The quencher absorbs the fluorescent signal via FRET (Fluorescence resonance energy transfer), but after trans-cleavage activity cleaves the short oligo, the fluorophore is separated from the quencher and can fluoresce freely. This increase in fluorescence is detectable by specialized machines, or the naked eye and UV flashlight if concentration of reporter oligos is high enough (Lau & Botella, 2017; Sun et al., 2021; Verosloff et al., 2019; Yuan et al., 2021). With this in mind, we aimed to create our own CRISPR-based detection platform for the prokaryotic plant pathogen group with rising attention, *Candidatus* Phytoplasma. Our work is intended to take the vast potential of CRISPR-based disease detection assay to boost the detection capacity of diagnostic researchers screening for pathogens, like phytoplasmas, by offering a strategy that could boost sensitivity of any Cas12a detection platform and could potentially one day be used for at-port and in-field screening (**Figure 1A**).

*Candidatus* Phytoplasmas are prokaryotic, phloem-limited, obligate biotrophs that live within host plant tissue or the salivary glands of insect vectors. Organisms in this group are particularly harmful due to the wide host range, diversity, and global prevalence (Bertaccini and Duduk, 2009; Kumari et al., 2019; Lee et al., 2000; Oshima et al., 2013). These organisms lack cell walls, which is the most obvious major distinction between mollicutes versus other prokaryotes like bacteria and archaea. Phytoplasmas are commonly vectored by ‘piercing-sucking’ insects, such as leafhoppers and whiteflies, that infect host plants when phytoplasmas in the salivary gland of the insects are transferred to the plant during phloem feeding (Du Toit, 2014; Krishnareddy, 2013) (**Figure 1B**). While in the phloem, phytoplasmas are believed to feed on nutrients from the plant host and multiply in number (Christensen et al., 2004; Zhao et al., 2014). The plant hosts will attempt to stop the spread by clogging up sieve plates in a process known as compartmentalization (Hogenhout et al., 2008). This strategy is able to stop the progression of further infestation through the phloem, however this also stifles the plant’s own phloem transport system. Phytoplasmas have been documented in over 600 unique plant host species, with many economically relevant crop species also being impacted and reporting even up to 100% yield loss in severe epidemics (Kumari et al., 2019; Rao and Kumar, 2017). The most visible signs present in highly infected plants are wilting, witches’ brooms, and phyllody. So far there has been no resistant cultivars to be found in any of the known susceptible species to phytoplasmas, except for one cultivar of jujube (Zhao et al., 2019). The most effective mitigation strategies for phytoplasma are to only propagate with confirmed-healthy plant material, exclude the insect vector if possible, and/or eliminate any individual hosts known to be infected with phytoplasmas to mitigate further spread to healthy individuals. Currently, the most recommended strategies to detect phytoplasma is to either perform a nested or semi-nested PCR followed by direct sequencing and matching with a known phytoplasma species or qPCR analysis (Christensen et al., 2004; Christensen el al., 2013; Hodgetts et al., 2009). Both strategies rely on meticulous techniques that require a trained analysist and expensive machinery.

Due to CRISPR being a highly modular technology, CRISPR-based detection assays have been heavily reprogrammed to target many other pathogens or sequences of interest across all kingdoms of species (Gootenberg et al., 2017; Li et al., 2023; Wei et al., 2023; Wheatley et al., 2021; Yin et al., 2022; Yuan et al., 2021). Much interest has been in expanding the usefulness of a CRISPR-based detection system by improving the methods to boost sensitivity via pre-amplification methods, intermediate reactions, or usage of microfluidic chips (Burkin et al., 2023; Cao et al., 2023; Li et al., 2024; Mao et al., 2024; Qiao et al., 2023; Wei et al., 2023; Wu et al., 2021; Yuan et al., 2023; Zhen et al., 2024). These methods have promised to be able to detect target DNA in concentrations ranging from nanomolar to even attomolar levels of concentration. However, few studies focus on the optimization of the CRISPR components and trans-cleaving reporter systems toward improved sensitivity. In this study, we aimed to improve CRISPR detection by focusing on these two CRISPR components. Since these are the common features of all CRISPR-based detection assays, focusing strictly on these two principles would better allow our technology to be implemented into any CRISPR-based detection platform wishing to push their sensitivity limits even further. We set out to determine if any Cas12a variants known to demonstrate improved genome editing could also demonstrate improved detection sensitivity, and if modifications to the standard reporter system would enhance Cas12a trans-cleavage signals (Ooi et al., 2021; Rossetti et al., 2022; Tong et al., 2021; Yan et al., 2019; Zhang et al., 2021). Here, we found that LbCas12a-Ultra outperformed all other Cas12a variants at most sites and combining with a 7nt stem-loop bulge on hairpin reporter oligos it led to a 10-fold improvement in detection sensitivity. We also implemented a multiplex strategy providing higher target coverage of more phytoplasma species than current “universal” phytoplasma detection methods. While these strategies were used to establish a robust detection platform for phytoplasmas, these strategies were designed to be applicable to any other CRISPR-Cas12a-based detection platforms.

## Results

### Designing of target sites

**Figure 2:**
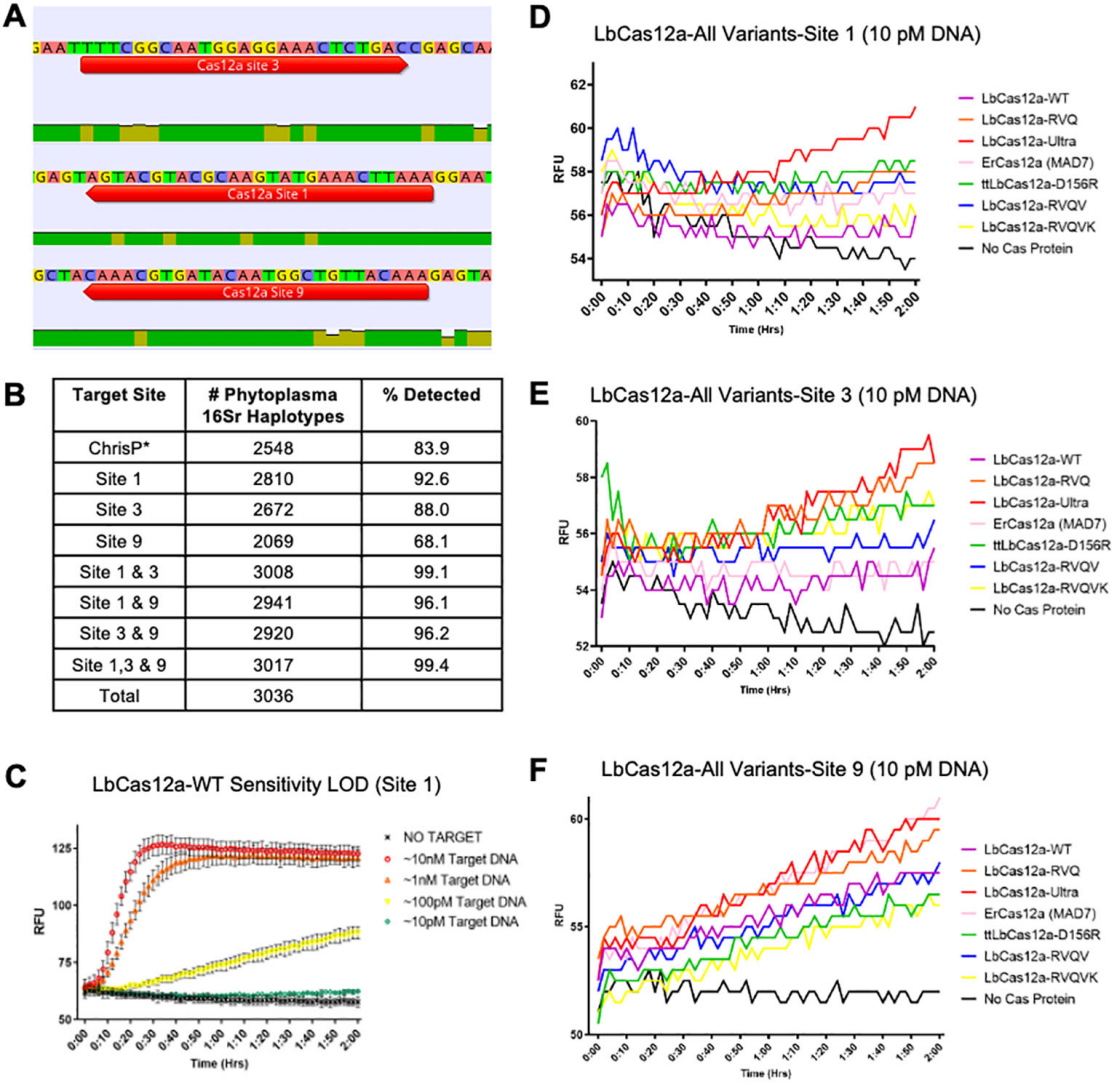
Exploring Cas12a orthologs and variants for high coverage and sensitivity detection of Phytoplasma. **(A)** Design of optimal Cas12a target sites for maximal detection of phytoplasma. Three target sites are selected based on a large-scale alignment of phytoplasma 16S genes. Green indicates =100% match across all sequences aligned; yellow indicates <100% match yet >30%; red (not pictured) indicates a <30% match. These specifications are presets from the Geneious Prime software. **(B)** Detection coverage by individual target sites and combination of multiple sites. The detection coverage of the universal primer set ‘ChrisP*’ is used as a control. **(C)** The Limit of Detection (LOD) established by the wild type LbCas12a at target site 1 for phytoplasma detection. RFU, Relative Fluorescence Units. **(D–F)** Comparison of Cas12a orthologs and LbCas12a variants for phytoplasma DNA detection at 10 pM concentration at site 1, site 3, and site 9. Three replicates were used for each data point.

There is an increasing diversity within the *Candidatus* Phytoplasma that can complicate detection systems. However, there is still some level of conservation across all phytoplasmas. The 16SrRNA gene is most often used to characterize new and identify existing species of prokaryotes. We used over 7,000 individual 16SrRNA (simply 16S, hereafter) genes of phytoplasmas as our working list of species and aligned to observe commonality. Across ~1500bp on the phytoplasma 16S gene, we were able to design 20 Cas12a crRNA sites that matched the criteria of containing a PAM-site (TTTV) and relatively high conservation for 20-23bp directly following (5’->3’). Each site was then screened for off-targeting by determining what sequences in other species would also be cleaved by the Cas12a proteins using that particular site. Targeting of a non-phytoplasma species in this sense will be considered off-targeting hereafter, since these would generate a false positive. Any sites that were believed to have off-targeting of a species that may be in close association with phytoplasma (plants, epiphytes, plant-fungal pathogens, etc.) were excluded and not considered to be a viable target site. After screening each site with this strategy, three target sites were believed to be optimal target sites for high on-targeting potential across many phytoplasma species with minimal off-targeting that may produce misleading results (**Figure 2A**; **Supplementary Table 1**). Next, we took these target sites and estimated the coverage we could capture across our entire working list of over 7,000 phytoplasma 16S gene sequences (**Figure 2B**). Cas12a typically can still get sufficient cleavage with 1-2 mismatches at a target site, even if there are mismatches are in the PAM site (J. S. Chen et al., 2018; Kaminski et al., 2021; Kleinstiver et al., 2019; Lu et al., 2022). The on-targeting potential of each site was compiled and checked for multiplexing potential with multiple target sites at the same time. These results were then compared to the coverage of a “universal probe” for qPCR characterized in Christensen et al., 2012 for determining the presence of phytoplasmas in suspected infected plant material (**Figure 2B**). Under the same perimeters as our screen for crRNAs, it is estimated that the Christensen qPCR probe could only capture 83.9% of phytoplasma species in our working list, while multiplexing our three crRNAs together could theoretically capture 99.4% of phytoplasma species documented on NCBI.

### Establishing CRISPR-based detection for phytoplasmas

Our first initial assay utilized LbCas12a-V4, a synthetic variant IDT developed that contains additional nuclear localization signals to boost *in vivo* editing efficiency in eukaryotes. Everything else about the protein is unchanged from the WT protein sequence and is thus considered our WT-LbCas12a protein for assay development. We used the standard report oligo, 5’6-FAM/TTATT/3’IABkFQ, from IDT. To establish the limit-of-detection (LOD) of this WT-LbCas12a-based detection assay, we followed closely a previous CRISPR-based detection protocol for Citrus Greening Disease (‘Huanglongbing’; HLB) (Wheatley et al., 2021). This was decided due to the pathogen that causes HLB (*Candidatus* Liberibacter *asiaticus*) occupies a similar ecological niche to phytoplasmas by living as an obligate biotroph in the phloem tissue of host plants (Fleites et al., 2014).

Maryland Pine Phytoplasma (MDPP) was first characterized in Costanzo et al. (2016). With PCR amplified MDPP genetic material, we developed our detection assays with the three crRNAs corresponding to the three chosen target sites. We found our assay generated rapid signaling saturation at high target DNA concentrations (10 nM and 1 nM) and linear signal was detected with 100 pM target DNA (**Figure 2C**). The assay could barely detect positive signals at 10 pM target DNA concentration (**Figure 2C**). This defined the limit of detection (LOD) with the current configuration and 10 pM is hence the baseline concentration for us to seek improvement on detection sensitivity.

### Determining the Cas12a variant with highest detection sensitivity

To enhance genome editing efficiency, previous studies explored Cas12a orthologs and engineered variants (Kleinstiver et al., 2019; Tang et al., 2017; Yan et al., 2019; Zhang et al., 2021). We hypothesized that certain Cas12a nucleases with enhanced genome editing activity may also confer higher sensitivity than WT-LbCas12a in a detection assay. To this end, we explored ErCas12a (MAD7) and five engineered LbCas12a variants: LbCas12a-D156R (also known as ttLbCas12a), LbCas12a-Ultra, LbCas12a-RVQ, LbCas12a-RVQV, and LbCas12a-RVQVK (Kleinstiver et al., 2019; Liu et al., 2020; Schindele and Puchta, 2020; Zhang et al., 2023; Zhang et al., 2022a). A cleavage assay was performed on gel electrophoresis to ensure compatibility with the same crRNA design on all variants (**Supplementary Figure 2**). From our LOD experiments with WT-LbCas12a, it was determined that ~10 pM was the LOD. Each Cas12a protein was tested for detection capacity at that same concentration, across all 3 of our target sites. At Phyto-16S Site 1 and 3, LbCas12a-Ultra outperformed all other variants at the same concentration of target DNA (**Figures 2D, E**). Consistent with their high genome editing efficiency, LbCas12a-D156R and LbCas12a-RVQ also showed high detection sensitivity (**Figures 2D, E**). At Phyto-16S Site 9, LbCas12a-Ultra and ErCas12a (MAD7) and outperformed other Cas12a proteins (**Figure 2F**). Overall, LbCas12a-Ultra demonstrated increased sensitivity over WT-LbCas12a and all other Cas nucleases at nearly every site. LbCas12a-Ultra was decided as the overall best performing Cas12a variant that we moved forward with for further optimization of the CRISPR-based detection platform.

### Non-linear report oligonucleotides for enhanced trans-cleavage activity

It was recently reported that a hairpin configuration of the reporter oligos outperformed standard linear reporters with higher detectable, trans-cleavage activity (Rossetti et al., 2022). A stem-loop bulge created by the hairpin configuration was believed to produce a more suitable structure for the enzymatic trans-cleavage activity of activated LbCas12a proteins. Either end contains nucleotides that will anneal to each other with repeated thymine(T) nucleotides in the middle to give the stem-loop bulge shape. Varying the number of T- repeats influenced the size of the stem-loop bulge, which was also shown to change the level of activity as well (Rossetti et al., 2022). However, only a limited number of stem-loop sizes were tested, and it was found the 10nt stem-loop reporter worked best (Rossetti et al., 2022).

**Figure 3:**
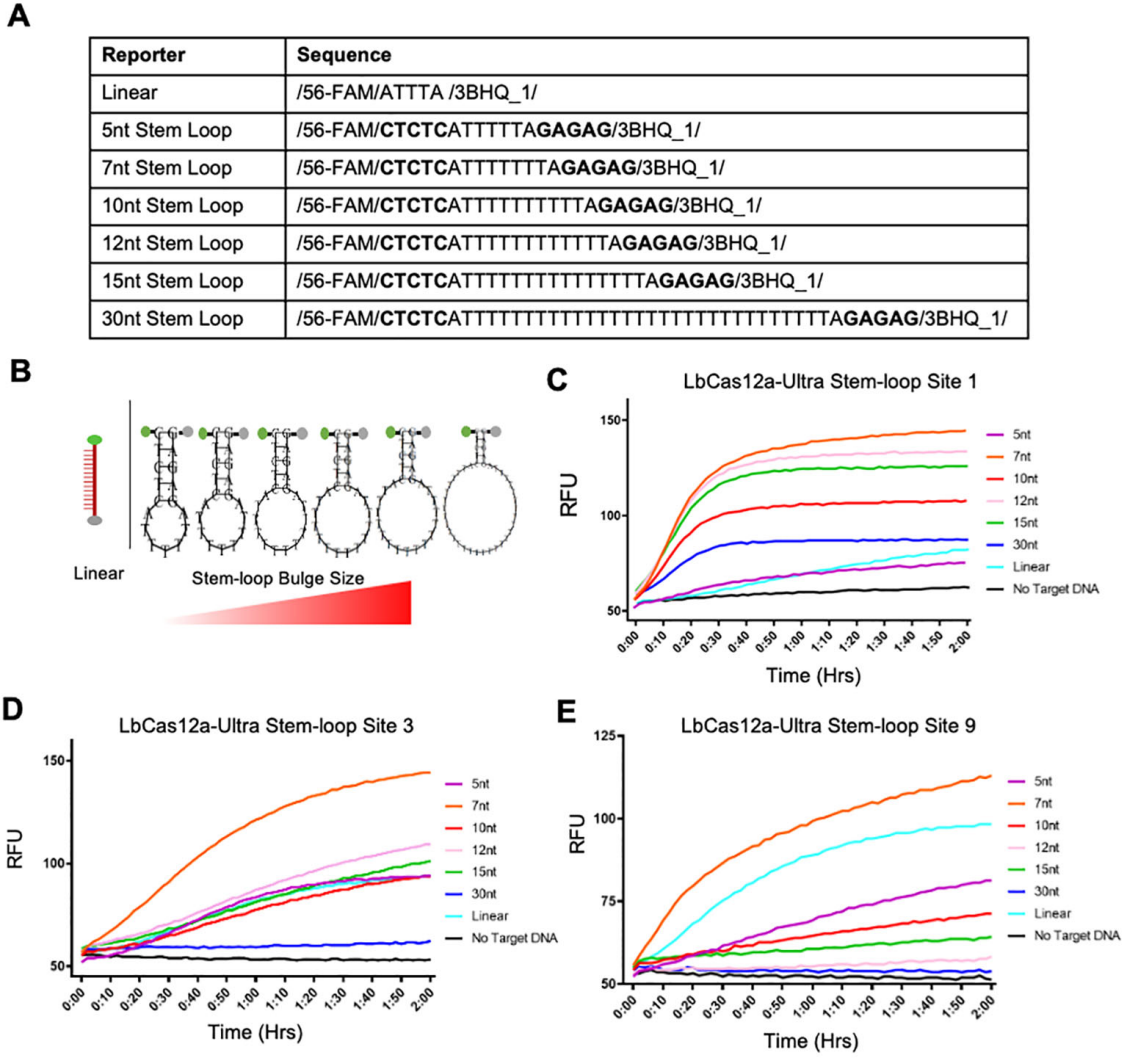
Further improvement of detection CRISPR-Cas12a-Ultra detection sensitivity by installing stem-loops of the reporter probes. **(A)** Sequences and configurations of different reporters. The standard linear reporters have most often been used in other detection platforms. Reporters with different sizes of hairpin stem loops are included for comparison. **(B)** Diagram showing the structures of different reporters. The vectorbuilder secondary structure tool (Gruber et al., 2015) was used to make stem-loop designs. Created with BioRender.com and VectorBuilder.com. **(C–E)** Comparison of different reporters for detection sensitivity of 100 pM DNA at three independent target sites with the optimal LbCas12a-Ultra. Three replicates were used for each data point.

Inspired by that study, we reasoned that the detection system with hairpin reporters could be improved by exploring more stem-loop sizes. Hence, we tested a series of stem-loop sizes of 5nt, 7nt, 10nt, 12nt, 15nt, and 30nt against the standard linear reporter (**Figures 3A, B**; **Supplementary Table 1**). Indeed, across all three tested target sites, the amount of RFU signal captured for 7nt stem-loop reporters was far higher than all other stem-loop reporters as well as the linear reporter (**Figures 3C–E**). This gives reason to believe that there was increased trans-cleavage activity from activated LbCas12a-Ultra proteins with 7nt stem-loop hairpins. The 7nt stem-loop reporter system was selected for all subsequent evaluation since this was our highest preforming reporter oligo across all target sites.

### Limit of detection with the optimized CRISPR-based detection system

**Figure 4:**
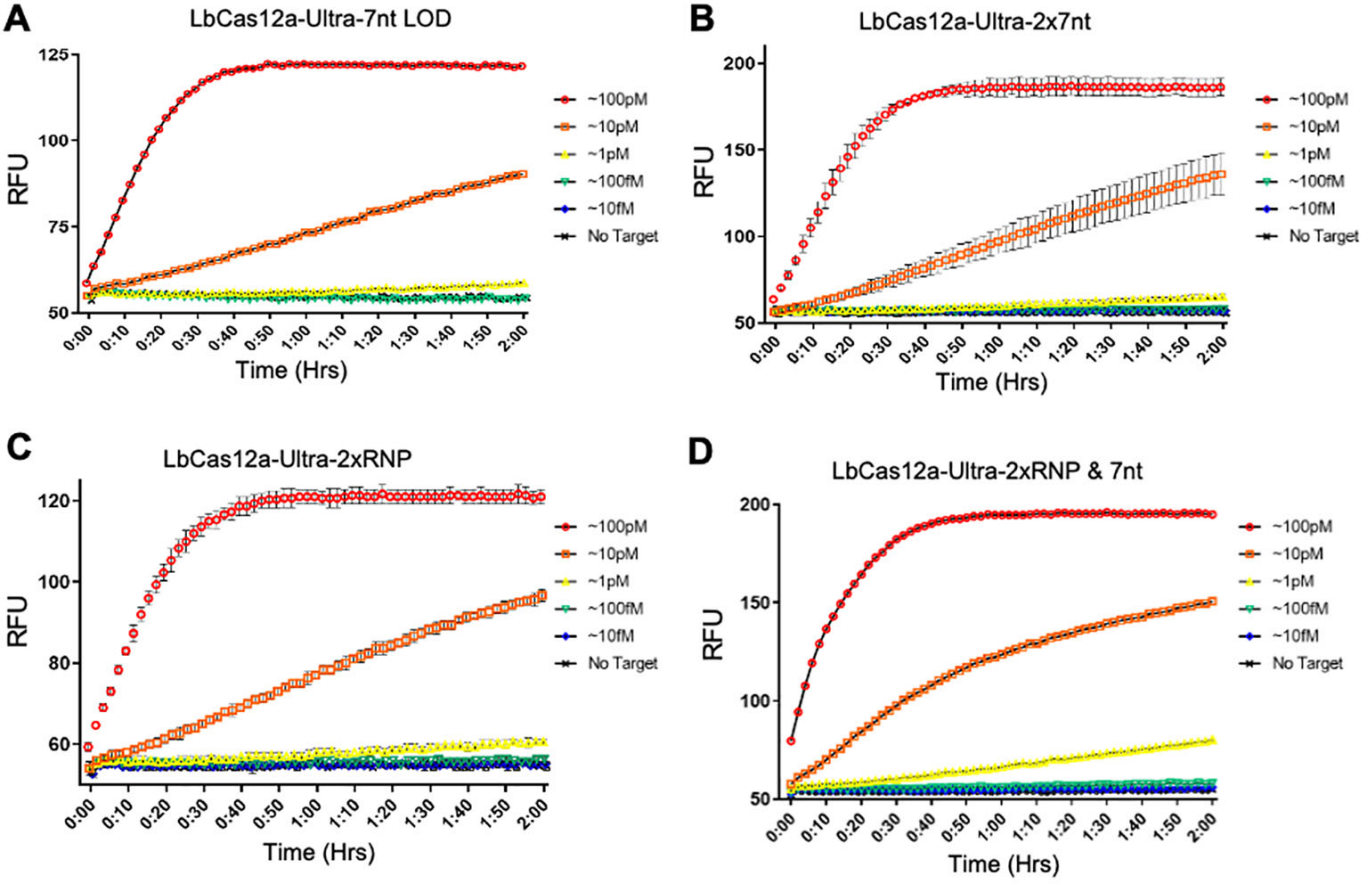
Doubling concentration of detection components for assay optimization. **(A)** Doubling the concentration of 7nt stem-loop reporter oligonucleotides (100 nM) did not significantly improve the limit of detection (LOD), but did increase single readout picked up by the fluorometer machine. **(B)** Doubling the RNP concentration (100 nM) did not improve LOD. **(C, D)** Doubling both 7nt stem-loop reporter (100 nM) or RNP (100 nM) showed slight improvement on LOD. Three replicates were used for each data point.

After determining the best performing Cas12a variant and the best performing reporter system, the next step is to determine if these optimized components improve the limit of detection (LOD) of a CRISPR-based detection platform. Additional log-dilutions were created down to ~100 fM concentration of target 16S DNA and assessed for our LOD using LbCas12a-Ultra and a hairpin with 7nt stem-loop bulge configuration of reporter oligos. With these two improvements to standard CRISPR-based detection assays, we were able to achieve detection levels down to ~1 pM concentration of target DNA on analytical samples (purified amplicon, **Figure 4A**). This is at least a 10-fold improvement over our standard LOD.

We sought further improvement by exploring additional optimization strategies for the detection system. We tried doubling the concentration of the 7nt stem-loop reporter, doubling the concentration of the Cas12a/crRNA RNPs, and simultaneous doubling both components (2x RNP & 7nt). Interestingly, none of the three approaches seemed to further improve the LOD sensitivity (**Figures 4B–D**). However, the consistent output of the detection suggests our newly established CRISPR detection system is very robust, less prone to the concentration variations of the major components.

**Figure 5:**
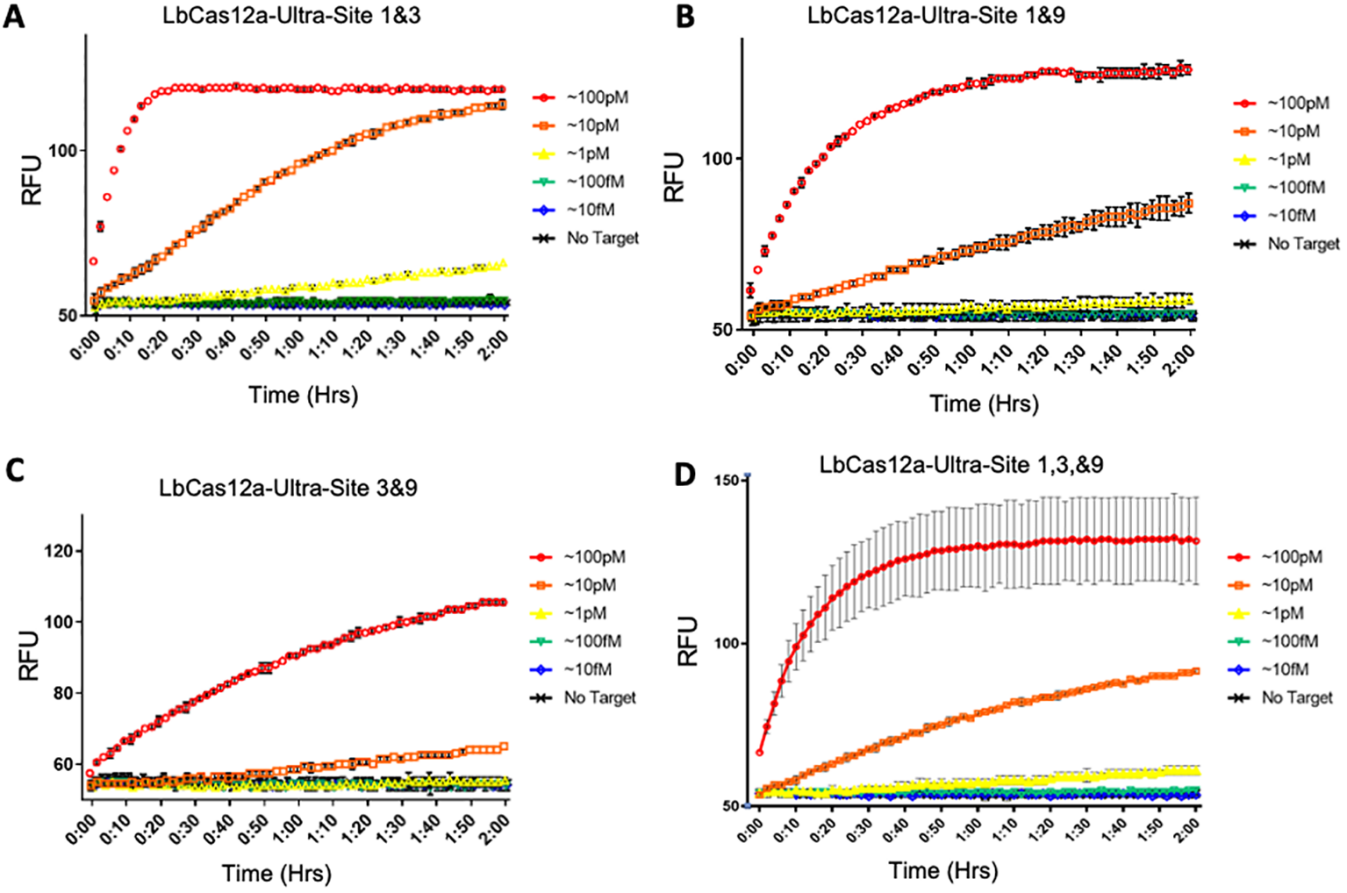
Assessment of detection sensitivity with multiplexed targeting sites. To observe if this optimization strategy would increase assay sensitivity at lower dilutions, assays were conducted with LbCas12a-Ultra and 7nt stem-loop reporter oligonucleotides by multiplexing Sites 1 & 3 **(A)**, Sites 1 & 9 **(B)**, Sites 3 & 9 **(C)**, and Sites 1, 3 & 9 **(D)**. Three replicates were used for each data point.

Based on our calculation, the use of all three crRNAs would allow for detection of 99.4% of all available phytoplasma genotypes from our working list (**Figure 2B**). We thus examined multiplexed detection using more than one crRNA to target more than one target site. All four multiplexed configuration of three target sites were explored. Although we did not observe any enhanced LOD with including two or three crRNAs in the assays, reliable detection output was observed with all crRNA or target site combinations (**Figures 5A–D**). This suggests that our detection system, in principle, can be used for simultaneous detection of multiple plant pathogens should we program our crRNAs specifically to these pathogens with a multiplexed detection setting.

### Evaluation against a panel of phytoplasma extractions and amplicons

**Figure 6:**
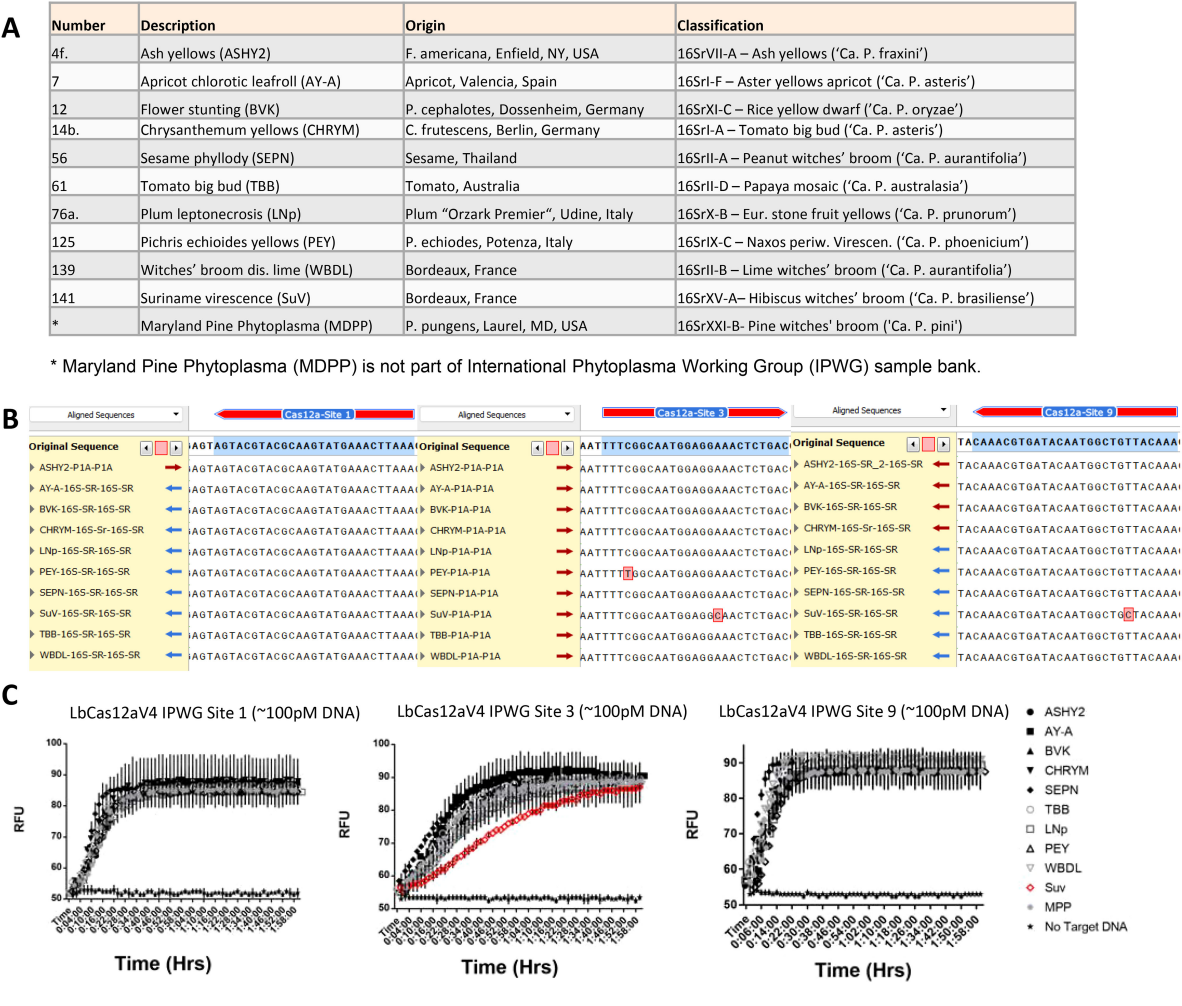
Assessment of inclusivity for the detection of diverse phytoplasma samples at three target sites with the optimized CRISPR-Cas12a system and PCR amplicons. **(A)** A list of 10 phytoplasma strains provided by the International Phytoplasma Working Group in Bologna, Italy. They represent a diverse panel across many branches of the 16S gene used to characterize phytoplasma. **(B)** Sequence alignment of target sites in the 16S genes of three different target sites in the select phytoplasma strains. These samples were all aligned to MDPP as consensus sequence. **(C)** Test of inclusivity of the three target sites for phytoplasma detection with wild type LbCas12a and linear reporters. SuV is highlighted at Cas12a-Phyto16S-Site3 to demonstrate a significant difference in detection capacity compared to all other phytoplasma samples. Three replicates were used for each data point.

Next, we wanted to evaluate the optimized detection system against a diverse panel of 10 additional phytoplasma samples obtained from the International Phytoplasma Working Group (IPWG), and these samples represent multiple different clades of diversification (**Figure 6A**; **Supplementary Figure 1**). These samples were confirmed phytoplasma positive via qPCR, and then amplified and sequenced following phytoplasma identification protocols (Christensen et al., 2004; Deng and Hiruki, 1991; Lee et al., 2004) (**Supplementary Figure 3**). These sequences were then aligned with our sequenced MDPP 16S gene as well as our designed target sites (**Figure 6B**). From our sequencing results it can be observed that most phytoplasmas have the identical sequences to target sites 1, 3 and 9, except the three discovered single nucleotide variations (SNVs) in the 16s rDNA sequences of Pichris echioides yellows (PEY) and Suriname virescence (SuV) (**Figure 6B**).

We conducted the detection assay tests of these phytoplasma amplicons at ~100 pM DNA concentration each. As expected, all phytoplasma amplicons were reliably detected with our assays using either of the three crRNAs for the three target sites. At target site 1, the same level of detection curves was observed among all phytoplasma samples (**Figure 6B**). Interestingly, although the target site 3 for the PEY sample contains a non-canonical TTTT PAM (vs the standard TTTV PAM) (**Figure 6B**), it did not cause significant signal compromise in detection (**Figure 6C**). Consistent with our result, non-canonical PAM sites were detected in Cas12a detection assays by others (Lu et al., 2022; Zhang et al., 2022b). The presence of one SNV relatively close to the seed sequence of the spacer at the target site 3 has reduced the detection sensitivity (**Figure 6B**), converting a non-linear curve to a near-linear curve as the signal output (**Figure 6C**). By contrast, the presence of on SNV outside of the seed sequence of the space in the SuV sample did not compromise the detection sensitivity at all (**Figures 6B, C**). Our results suggest that the impact of mismatch mutations in the spacer regions is dependent on their relative positions, similar to what has been observed in Cas12a-mediated genome editing experiments (Chen et al., 2018; Kleinstiver et al., 2019; Ooi et al., 2021; Swarts and Jinek, 2019; Yan et al., 2019; Zhang et al., 2021; Molina Vargas et al., 2023).

**Figure 7:**
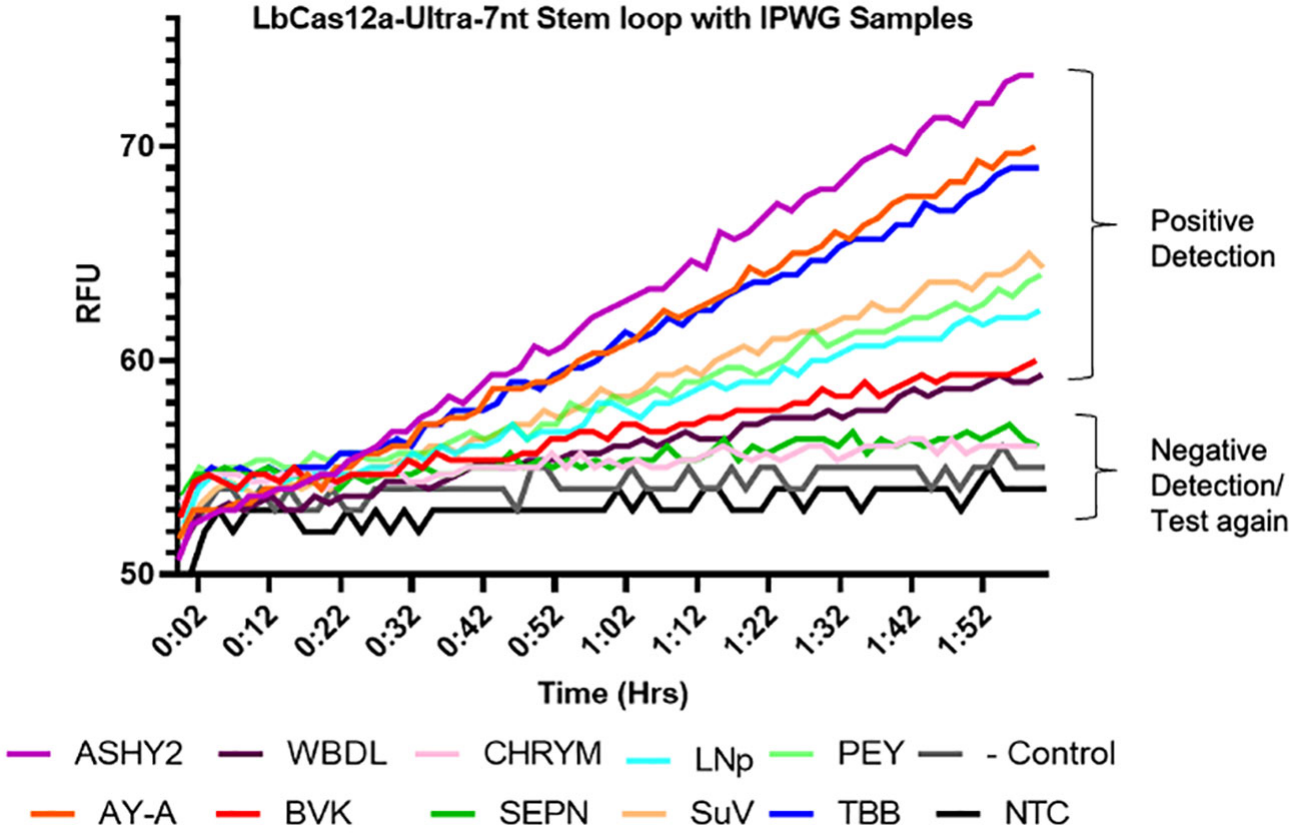
Amplification-free detection of a diverse phytoplasma samples with the optimized CRISPR-Cas12a system. Phytoplasma samples inoculated into an alternative host (*Vinca minor*, periwinkle) across a diverse panel of subgroups were tested with the optimized CRISPR-Cas12a detection system. LbCas12a-Ultra, 7nt stem-loop reporter oligonucleotides, and multiplexing all three target sites were used together for this assay. Three replicates were used for each data points.

To further evaluate our CRISPR-based detection assay, we then attempted to detect the phytoplasma samples directly from the DNA extractions we obtained from IPWG with no pre-amplification. These extractions came from periwinkle (*Vinca minor*) tissue infected with the particular phytoplasma species. A negative control was also used, which was pine tree tissue confirmed to be phytoplasma-free via qPCR, since our positive control, MDPP, is extracted from infected pine. Our results demonstrated that we could detect most phytoplasma samples (**Figure 7**). Out of 11 phytoplasma samples, eight were detected by multiplexed detection of all three target sites, and two samples (CHRYM and SEPN) were barely detected, only acquiring slightly higher RFU signals above the NTC samples, not enough to determine a true positive (**Figure 7**). Failed detection of the three phytoplasma samples could be due to that these samples contained less phytoplasma DNA titer in extracted plant material than other extracted tissues. Nevertheless, these tests demonstrated that our target sites have great coverage of a diverse set of phytoplasmas, and we were able to detect phytoplasmas directly from plant DNA extractions containing phytoplasmas.

### Establishment of lateral flow assay compatibility

**Figure 8:**
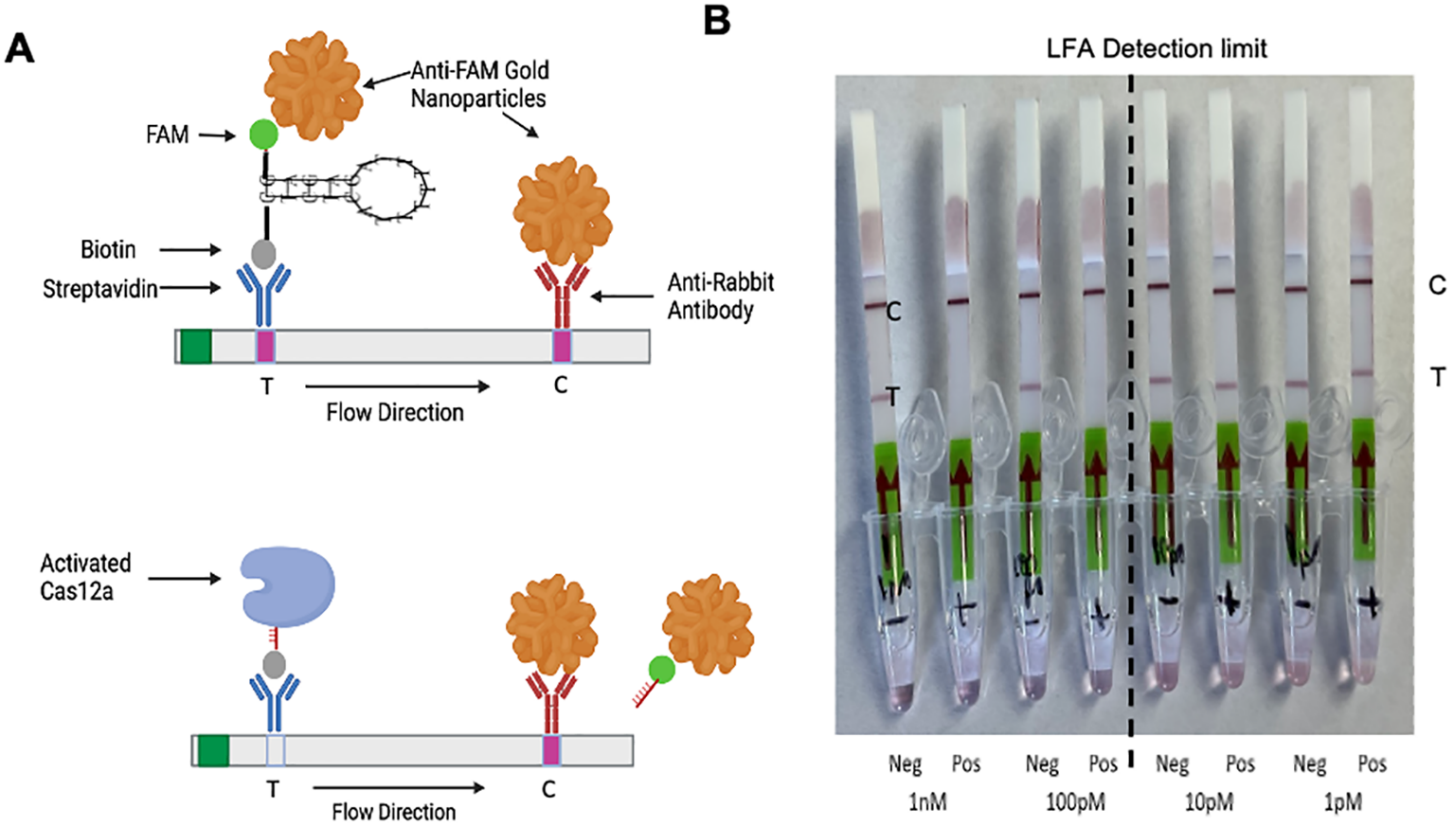
The optimized CRISPR-Cas12a configuration is compatible for phytoplasma detection by lateral flow assay (LFA). **(A)** Diagram of LFA, where absence of signal for the ‘T’ band indicates positivity. This is due to trans-cleavage activity separating biotin from FAM on the same oligo. The FAM, still attached to Anti-FAM gold nanoparticles, flow uninhibited up the LFA paper strip. The vectorbuilder secondary structure tool (Gruber et al., 2015) was used to make stem-loop designs. Created in https://BioRender.com. **(B)** LFA-based detection of the phytoplasma at different DNA concentrations. When using the most optimized system of LbCas12a-Ultra, 7nt stem-loop reporter oligo, and 3 target sites multiplexed, the LOD is between 100 pM and 10 pM of target DNA. This assay could be done in-field for much faster turnover at testing sites.

To evaluate the potential for this CRISPR detection assay for on-site detection of Phytoplasmas on healthy or infected plant material, we also developed a lateral flow assay (LFA) strategy based on our optimized LbCas12a-Ultra and 7nt stem-loop probe. LFA assays use engineered paper test strips to allow the results to be visualized by the naked eye, which is commonly used for at-home pregnancy tests or nowadays rapid Covid-19 antigen tests. Our CRISPR-Cas12a based LFA was developed based on the HybriDetect-Universal LFA strips and an oligo reporter conjugated with FAM and biotin at both ends, respectively. In the absence of target pathogen DNA, the gold-particle conjugated anti-FAM antibody will be sequestrated to both the “Test Band” site and the “Control Band” site, resulting in two positive bands (**Figure 8A**, upper panel). Counterintuitively, such double-positive bands indicate a negative test result. In the presence of the target pathogen and when the probe is sufficiently cleaved due to the on-target binding-triggered trans-cleavage activity of LbCas12a, the signal at the “T Band” band will disappear and only the “Control Band” will show signal due to capturing the anti-FAM antibody at this position (**Figure 8A**, lower panel). This loss of signal at the “T” line would indicate a positive detection, a “one-band positive strategy”. This may juxtapose other assays that use one band to indicate negative and two bands to indicate positive, a “two-band positive” strategy (Lei et al., 2023; Wheatley et al., 2022; Yuan et al., 2023). The principles of the “two-band strategy” rely on oversaturating the first, lower band with reporter oligos to capture all the anti-FAM gold nano-particles so that any reporter degradation will result in a color change of the second, higher band. This “one-band strategy” used here, based on its design principle, will give higher confidence in a true positive result. Based on this strategy, the absence of a band should only occur with high saturation of cleaved reporter oligos, whereas partial degradation of reagents could lead to a false positive with the “two-band positive strategy”.

Based on this principle, we tested our LFA system with a series of target DNA concentrations from PCR amplicons of MDPP 16S gene. We found the LFA assay could reliably detect target DNA concentrations at 1 nM and 100 pM (**Figure 8B**). When the target DNA concentration was reduced to 10 pM and 1 pM, signals of “T” line showed up (**Figure 8B**). These data suggest the LOD of our LFA is between 10 pM and 100 pM. The reason why the LOD appears to be much higher for LFA compared to our fluorescence-based testing is because there needs to be full saturation of trans-cleaved reporter oligos and the signal readout is rather qualitative than quantitative (**Figure 8A**). While LFA is not as sensitive or quantitative as fluorescence-based tests, it is a much more portable, easy-to-use, and simple test that could be performed on-site with only just incubation at 37°C, which can even be done with human body heat.

## Discussion and conclusion

Our main goal of this research was to create a new CRISPR-based plant pathogen detection platform that did not require pre-amplification by improving the CRISPR components used for CRISPR-based detection. Most CRISPR-based detection platforms optimize pre-amplification strategies or use unique alternatives to boost sensitivity (Burkin et al., 2023; Shi et al., 2021; Wu et al., 2021; Yang et al., 2023; Zhu et al., 2020). While this does help to achieve a higher sensitivity, these assays often can lead to false positives or misleading results. However, there are few examples of improvements on only the CRISPR components alone (Chen et al., 2023; Lu et al., 2022; Xue et al., 2022; Yang et al., 2024). Based on our results, we demonstrated exceptional improvements over the standard CRISPR-based detection platform and allowed for sensitive yet specific detection of a diverse panel of 11 phytoplasma species. We screened multiple Cas12a nucleases that have shown remarkable improvements in gene editing efficiency and scope and found multiple Cas12a nucleases outperformed the WT-LbCas12a (**Figures 2D–F**). Among them, LbCas12a-Ultra was the best-performing Cas12a variant, far exceeding the performance of the WT-LbCas12a. Since most detection platforms utilize the WT-LbCas12a, these developed assays may increase their detection sensitivity far greater with integration of LbCas12a-Ultra (Cai et al., 2022; Liu et al., 2022; Wang et al., 2023b; Xu et al., 2020). LbCas12a-Ultra is a variant discovered by IDT through mutagenized protein evolution. Our data clearly suggest that those Cas12a nucleases with high performance in genome editing can be harnessed for enhancing Cas12a-mediated detection technologies.

We also showed that compared to linear reporters most commonly used Cas12a detection platforms, hairpin reporters with a stem-loop configuration, particularly with a 7nt stem-loop bulge, confer enhanced detection sensitivity. It is believed that this hairpin, stem-loop configuration produces a better steric shape for the activated Cas12a trans-cleavage activity. This improves the enzymatic activity of these proteins, which is translated into increased detection sensitivity of the assay. However, the original research that reported the use of hairpin reporters suggested that 10nt stem-loop budge is the best configuration (Rossetti et al., 2022). Hence, our research demonstrated further improvement of the Cas12a-based detection systems using hairpin reporters by discovering a 7nt stem-loop bulge to be an even more optimal shape.

By combining our best-performing LbCas12a-Ultra variant, and our best-performing 7nt stem-loop reporters, we were able to achieve a 10-fold increase in sensitivity compared to the standard detection assay that uses WT-LbCas12a and linear reporters. We believe these are very interchangeable innovations that could be applied to improve any Cas12a-based detection systems with higher sensitivity. For example, stem-loop reporters could be easily integrated into most detection platforms that rely on fluorescence-based readings to boost their detection sensitivity, like Cas12a detection assays that utilize micro-fluidic chips or other unique shapes of the reporter oligo/readout strategy (Bao et al., 2023; Burkin et al., 2023; Chen et al., 2023; Puig-Serra et al., 2022; et al., 2023b; Wang et al., 2023a; Wu et al., 2021).

In this study, we did not use a pre-amplification step. Without pre-amplification, it is possible that our optimized CRISPR-Cas12a detections assays may still fall short in detecting pathogens from all infected plants including phytoplasma-infected tissues, especially when the pathogen titers are low. However, it is envisioned that our CRISPR-Cas12a based detection system, when combined with a pre-amplification strategy, may confer higher detection sensitivity. Also, our detection platform may need some additional improvements to be implemented in real plant pathogen screening practices. For example, in the case of phytoplasma detection, these improvements include but are not limited to: further validation with additional phytoplasma samples, off-target screening of unrelated species, and even great improvements to the LOD to detect very low titer phytoplasma infected samples. With continued improvements, CRISPR-based detection methods are promising tools for pathogen detection and beyond.

## Materials and methods

### Designing target sites

Since the 16SrRNA gene is most often used to characterize new and identify existing species of prokaryotes we decided upon this gene sequence for our target site. This was assumed to give us the greatest ability to determine our coverage of multiple phytoplasma species. We pulled over 13,000 individual 16SrRNA (simply 16S, hereafter) genes of phytoplasma uploaded to NCBI-BLAST using Geneious Prime software. We removed duplicates and sequences seeming to be incorrect characterizations on NCBI, such as species mislabeling or the sequence for a different gene. The resulting 7,000+ reliable 16S sequences were used as our working list of phytoplasma species and aligned to observe commonality. From these large alignments using MUSCLE, we searched first for PAM sites (5’-TTTV-3’/or 3’-BAAA-5’ for PAM sites on the complimentary strand) that also shared high levels of sequence complimentary downstream to the PAM. Across ~1500bp on the phytoplasma 16S gene, we were able to design about 20 potential Cas12a crRNA sites. Each site was then screened on NCBI-BLAST, excluding phytoplasmas, to determine what sequences in other species would be cleaved by the Cas12a proteins using that particular site. Successful targeting of a non-phytoplasma species in this sense will be considered “off-targeting” hereafter. Any sites that were believed to have off-targeting of a species that may be in close association with phytoplasmas (plants, epiphytes, plant-fungal pathogens, etc.) were excluded and not considered to be a viable target site. After screening each site via these steps, three target sites were identified for high on-targeting potential across many phytoplasma species with minimal off-targeting.

To then measure the coverage of our proposed target sites on our entire working list we performed a probe coverage analysis. This is similar to measuring the coverage a primer set might have on a large population. We specified to only screen for unique sequences. This parameter is meant to exclude repeated sequence submissions of 100% identical sequence similarity and reduce bias in coverage from these repeats. These specifications resulted in testing our coverage across 3036 unique phytoplasma ‘haplotype’ strains from the original working list of over 7000+ phytoplasma 16S gene sequences.

### Maryland pine phytoplasma DNA preparation and validation

DNA was extracted using Qiagen DNeasy Plant Pro Kit on suspected infected pine tissue provided by USDA-APHIS, PPCDL (Laurel, MD, USA). Phytoplasma 16S DNA was amplified from extracted DNA using a semi-nested PCR technique. The first PCR used primers P1 and 16S-SR (10 uM), and Platinum™ Taq DNA Polymerase kit (Invitrogen). The cycling conditions were: 95°C for 5 min; 37x (95°C for 15s; 55°C for 30s; 72°C for 1.5 min); 72°C for 4 min. A 1:30 dilution was performed on the amplified material of the first round of PCRand used as the template for the second round of PCR. The primer set used for the second round are P1A and 16S-SR with the same cycling conditions. A PCR clean-up is performed following QIAquick PCR Purification Kit (Qiagen, Germany) and verified by Sanger sequencing. Primers P1A and 16S-SR are also used as sequencing primers (**Supplementary Table 2**). Amplified DNA concentrations was quantified using Nanodrop reader.

### Cas12a nuclease preparation

The Cas12a recombinant proteins were produced at Integrated DNA Technologies (IDT, Coralsville, IA, USA). Briefly, DNA sequences encoding LbCas12a proteins with C-terminal 6 x His-tagged were cloned into pET28a vector and transformed into *E. coli* BL21(DE3) cells (EMD Millipore). The transformed DE3 cells were grown in TB medium with 50 µg/mL Kanamycin. When OD_600_ reached 0.6-0.8, the cells were chilled at 4°C for 30 minutes, and IPTG was added to 1 mM concentration to induce protein expression at 4°C for 12-16 hours. Then, cells were harvested by centrifugation (4000xg, 20 minutes, 4°C), and resuspended in the lysis buffer (20 mM NaPO_4_, pH 6.8, 0.5M NaCl, 15 mM Imidazole, 10 mM CaCl_2_, and 10% Glycerol) with addition of protease inhibitor cocktail (Sigma: 11873580001), DNaseI and Lysozyme. The lysed resuspended cells were passed through Avestin Emulsiflex C3 three times at 15,000 psi, 4°C. The cell lysate was further centrifuged at 14,000xg for 40 minutes, and the soluble fraction was purified by Nickle affinity (HisTrap HP, 5 mL, Cytiva) and cation exchange chromatography (HiTrap Heparin, 5 mL, Cytiva). Subsequently, purified protein was concentrated (Amicon centrifugal filter, 10 kDa), and dialyzed against storage buffer overnight (20 mM TrisHCl, pH 7.4, 0.3M NaCl, 0.1 mM EDTA, 50% Glycerol, and 1 mM DTT). The protein concentration was measured by Nanodrop using an extinction coefficient at 167,780 M^-1^cm^-1^. A protein alignment of LbCas12a variants used in this study was provided as **Supplementary Figure 4**.

### CRISPR-based detection for MDPP

Reporter oligos (5’6-FAM/TTATT/3’IABkFQ) were also ordered from IDT. The concentration of each component was diluted and made into a master mix so that 45 μL of final reaction volume would contain: 50 nM of LbCas12a-V4, 62.5 nM of crRNA, 50 nM of reporter oligos, and 1x NEB CutSmart Buffer (New England Biolabs, Ipswich, MA, USA). 45 μL of mixed components were added to a Nunc 96-well optical bottom plate. Amplified 16S gene from the MDPP phytoplasma was diluted in a log series from ~100 nM- ~100 pM concentration of DNA. 5 μL of PCR amplicons of 16S gene from MDPP was added to each well to bring a final volume of 50 μL in each well, also giving each target DNA spike final concentration an additional 10-fold dilution upon insertion to well (~10 nM- ~10pM). The 96-well plate was loaded into a BioTek Synergy HTX Multimode Plate Reader and set to run for 2 hours at 37° C taking a fluorescence reading every 2 minutes. Readings from the machine were collected and analyzed to determine the LOD for our detection assay. The machine was set to standard dynamic range with a gain setting of 35. The EX/EM was 485/20 nm; 525/20 nm. NTC samples were used in every test for test validation. NTC sample wells contained all the same reagents as any other well in the same concentrations, and the only difference was the absence of target DNA. A positive detection was determined by a signal of RFU higher than the NTC. Generally, NTC would get RFU signal ~52-54, any signal was considered a positive if the RFU was ≥2 RFU from the max RFU of the NTC. This same procedure was followed when non-linear reporters were tested. For further optimization of the assay, different concentrations of Cas12a protein and reporters as well as the combination of more than crRNAs were used without altering other reaction components and the final volume.

### Evaluation with a panel of phytoplasma extractions

The 10 additional phytoplasma samples were ordered from the International Phytoplasma Working Group (https://www.ipwgnet.org, Bologna, Italy) across multiple different clades of diversification of 16S gene phytoplasma groups. Samples were hydrated with molecular grade water to reach 20 ng/μL each, based on the instruction of the provider. However, it was impossible to quantify how much of the DNA in the sample was truly phytoplasma DNA mixed in with DNA from the plant host and other microbes. These samples were amplified and sequenced using the same protocol to identify and sequence the MDPP samples. These sequences were then aligned with our sequenced MDPP 16S gene as well as our crRNA designed target sites. WT-LbCas12a(V4) and standard linear reporters (/56-FAM/ATTTA/3BHQ_1/) were used in the initial validation tests. To further validate phytoplasma samples from IPWG, an optimized CRISPR assay was applied. The reaction contained 50 nM of LbCas12a-Ultra, 62.5 nM of crRNA (split equally between each target site), 50 nM of 7nt stem-loop reporter oligos, 5 μL phytoplasma rehydrated extraction, and 1XNEB CutSmart Buffer. As a negative control, we used extracted DNA from a confirmed healthy pine tree. MDPP sample was used as a positive control.

### Establishment of lateral flow assay compatibility

Our CRISPR-Cas12a based LFA was developed based on the HybriDetect-Universal LFA strips (Milenia Biotec, Gießen, Germany). The reporter utilizes FAM on the 5’-end of the reporter oligo, but the other end contains a biotin molecule on the 3’-end. Cleavage of the reporters by Cas12a’s *trans*-cleavage nuclease activity result in target DNA detection. The basic principle is based on the detection of the gold particles coated with anti-FIFC/FAM antibody. A loss of the visualization of the ‘Test line’ will suggest positive detection of target DNA. To adapt our optimized CRISPR-based detection system to an LFA assay, we first redesigned our best-performing 7nt stem-loop reporter oligos to be LFA compatible, which contain biotin at the 3’-end as opposed to 3’-BHQ (/56-FAM/CTCTCATTTTTTTAGAGAG/3Biotin/) (IDT, Coralsville, IA, USA). The reaction was performed in PCR tubes with a volume of 20 μL. Otherwise, all other components from the assay were kept the same from fluorescence-based detection (50 nM LbCas12a-Ultra, 62.5 nM crRNA, 1XNEB CutSmart Buffer, 2 hours at 37°C). Target DNA was serially log-diluted just like our LOD screening tests in earlier technology development from 1 nM to 1 pM. After the reaction time, samples were removed, then LFA buffer was added and mixed per provided protocol from the manufacturer. Hybridetect- Universal LFA test strips were added to tubes and observed after 10 min to see if successful detection had occurred. Non-template control (NTC) tubes containing no target DNA were made for each spike DNA concentration in order to compare the test bands to determine at what concentration(s) are the bands between a positive and negative tube producing indistinguishable identical bands at the “Test Band” site.

## Data Availability

The datasets presented in this study can be found in online repositories. The names of the repository/repositories and accession number(s) can be found in the article/**Supplementary Material**.
