# Supplementary material for: Amplification-free detection of plant pathogens by improved CRISPR-Cas12a systems: a case study on phytoplasma
Source: Front Plant Sci. 2025 Mar 6;16:1544513. doi: 10.3389/fpls.2025.1544513 (PMC11924941; doi:10.3389/fpls.2025.1544513)
Supplement: Supplementary file 1 [file Table1.docx]

**Supp. Table 1. crRNAs and Reporter Oligo Sequences**

| **Target** | **gRNA/ Reporter Oligo** | **Sequence** |
| --- | --- | --- |
| **Phytoplasma 16S Gene** | **Cas12a-Site 1 (RNA)** | 5’- rArGrUrUrUrCrArUrArCrUrUrGrCrGrUrArCrGrUrArCrU - 3’ |
|  | **Cas12a-Site 3 (RNA)** | 5’- rGrGrCrArArUrGrGrArGrGrArArArCrUrCrUrGrArC - 3’ |
|  | **Cas12a-Site 9 (RNA)** | 5’- rUrArArCrArGrCrCrArUrUrGrUrArUrCrArCrGrUrUrUrG - 3’ |
| **Reporter Oligos** | **Linear Reporter (Fluro)** | 5’-/56-FAM/ATTTA /3BHQ_1/-3’ |
|  | **Linear Reporter**  **(LFA)** | 5’-/56-FAM/ATTTA /3Bio1/-3’ |
|  | **5nt Stem Loop (Fluro)** | 5’-/56-FAM/**CTCTCA**TTTTT**AGAGAG**/3BHQ_1/-3’ |
|  | **7nt Stem Loop (Fluro)** | 5’- /56-FAM/**CTCTCA**TTTTTTT**AGAGAG**/3BHQ/ -3’ |
|  | **7nt Stem Loop (LFA)** | 5’- /56-FAM/**CTCTCA**TTTTTTT**AGAGAG**/3Bio/ -3’ |
|  | **10nt Stem loop (Fluro)** | 5’-/56-FAM/**CTCTCA**TTTTTTTTTT**AGAGAG**/3BHQ_1/-3’ |
|  | **12nt Stem loop (Fluro)** | 5’-/56-FAM/**CTCTCA**TTTTTTTTTTTT**AGAGAG**/3BHQ_1/-3’ |
|  | **15nt Stem Loop (Fluro)** | 5’-/56-FAM/**CTCTCA**TTTTTTTTTTTTTTT**AGAGAG**/3BHQ_1/-3’ |
|  | **30nt Stem Loop (Fluro)** | 5’-/56-FAM/**CTCTCA**TTTTTTTTTTTTTTTTTTTTTTTTTTTTTT  **AGAGAG**/3BHQ_1/-3’ |

**Supp. Table 2. Primers and Probes (PCR & qPCR)**

| **Target** | **Primer/Probe** | **Sequence** |
| --- | --- | --- |
| **Phytoplasma 16S Gene**  **(PCR/Sequencing)** | **P1** | **5’- AAGAGTTTGATCCTGGCTCAGGATT - 3’** |
|  | **P1A** | **5’- AACGCTGGCGGCGCGCCTAATAC- 3’** |
|  | **16S-SR** | **5’- GGTCTGTCAAAACTGAAGATG- 3’** |
| **Phytoplasma 23S Gene**  **(qPCR)** | **JH-F1** | **5’- GGTCTCCGAATGGGAAAACC-3’** |
|  | **JH-F all** | **5’- ATTTCCGAATGGGGCAACCC-3’** |
|  | **JH-R** | **5’-CTCGTCACTACTACCRGAATCGTTATTAC** |
|  | **JH-P uni** | **5’-/FAM/AACTGAAATATCTAAGTAAC/MGBNFQ/-3’** |
|  | **18S-uni F** | **5’-GACTACGTCCCTGCCCTTTG-3’** |
|  | **18S- uni R** | **5’-AACAYTTCACCGGAYCATTCA-3’** |
|  | **18S- uni P** | **5’-/ABY/ACACACCGCCCGTCGCTCC/QSY/-3’** |

**Suppl. Sequences- Phytoplasma 16S genes**

**>MK089819_(2) Candidatus Phytoplasma pini isolate PineBLD 16S ribosomal RNA gene, partial sequence**

GAACGACTGCTAAGACTGGATAGGAATAAAAAGGCATCTTTTTATTTTTTAGAGACCTTTTTCGAAAGGTATGCTTAAAGAGGGGCTTGCGCCATATTAGTTAGTTGGTAGGGTAAAAGCCTACCAAGACGATGATATGTAGCTGGACTGAGAGGTTGAACGGCCACATTGGGACTGAGACACGGCCCAAACTCCCACGGGAGGCAGCAGTAGGGAATTTTCGGCAATGGAGGAAACTCTGACCGAGCAACGTCGCGTGAACGATGAAGTATTTCGGTATGTAAAGTTCTTTTATTGAAGAAGAAAAAATAGTGGAAAAACTATCTTGACGCTATTCAATGAATAAGCCCTGGCTAATTATGTGCCAGCAGCCGCGGTAATACATAAAGGGCGAGCGTTATCCGGAATTATTGGGCGTAAAGGGTGCGTAGGCGGTTTGTTAAGTCTTTAGTTTAATCTCAGTGCTTAACGCTGTTCTGCTAGAGAAACTGACTTACTAGAGTGAGATAGAGGCAAGTGGAATTCCATGTGTCAGCGGTAAAATGCGTAAATATATGGAGGAACACCAGAGGCGTAGGCGGCTTGCTGGGTCTTTACTGACGCTGAGGCACGAAAGCGTGGGGAGCAAACAGGATTAGATACCCTGGTAGTCCACGCCGTAAACGATGAGTACTAAGTGTCGGGGAAACTCGGTACTGAAGTTAACACATTAAGTACTCCGCCTGAGTAGTACGTACGCAAGTATGAAACTTAAAGGAATTGACGGGATCCCGCACAAGCGGTGGATCATGTTGTTTAATTCGAAGATACACGAAAAACCTTACCAGGTCTTGACATACTCTGCAAAGCTGTAGAAATACAGTGGAGGTTATCAGGGATACAGGTGGTGCATGGTTGTCGTCAGCTCGTGTCGTGAGATGTTAGGTTAAGTCCTAGAACGAGCGCAACCCTTGTCGTTAGTTACCAGCACGTTATGGTGGGCACTTTAACGAGACTGCCAATGAAATATTGGAGGAAGGTGAGGATTACGTCAAATCATCATGCCCCTTATGATCTGGGCTACAAACGTGATACAATGGCTGTTACAAAGAGTAGCTGAAACGTGAGTTTTTGGCCAATCTCAAAAAAACAGCCTCAGTTCGGATTGAAGTCTGCAACTCGACTTCATGAAGTTGGAATCGCTAGTAATCGCAAATCAGCATGTTGCGGTGAATACGTTCTCGGGGTTTGTACACACCGCCCGTCA

**>AY858053 Candidatus Phytoplasma aurantifolia 16S ribosomal RNA gene, partial sequence**

TTTTAAAGACCTAGTAATAGGTATGCTTTAGGAGGGGCTTGCGCCATATTAGTTAGTTTGTAGGGTAATGGCCTACCAAGACGATGATGTGTAGCTGGACTGAGAGGTTGAACAGCCACATTGGGACTGAGACACGGCCCAAACTCCTACGGGAGGCAGCAGTAGGGAATTTTCGGCAATGGAGGAAACTCTGACCGAGCAACGCCGCGTGAATGACGAAGTACTTCGGTATGTAAAGTTCTTTTATCAAGGAAGAAAAGCAAATGGCGAACCATTTGTTTGCCGGTACTTGATGAATAAGCCCCGGCTAATTATGTGCCAGCAGCCGCGGTAAGACATAAGGGGCAAGTGTTATCCGGAATTATTGGGCGTAAAGGGTGCGTAGGCGGTCTAGTAAGTCAGTGGTGTAATGGCAACGCTTAACGTTGTCCGGCTATTGAAACTGCTAAACTTGAGTTAGATAGAGGCGAGTGGAATTCCATGTGTAGCGGTAAAATGCGTAAATATATGGAGGAACACCAGAGGCGTAGGCGGCTCGCTGGGTCTTAACTGACGCTGAGGCACGAAAGCGTGGGGAGCAAACAGGATTAGATACCCTGGTAGTCCACGCCGTAAACGATGAGTACTAAGTGTCGGGTTAAACCGGTACTGAAGTTAACACATTAAGTACTCCGCCTGAGTAGTACGTACGCAAGTATGAAACTTAAAGGAATTGACGGGACTCCGCACAAGCGGTGGATCATGTTGTTTAATTCGAAGATACCCGAAAAACCTTACCAGGTCTTGACATGTTTTTGCAAAATGATAGTAGTATCGTGGAGGTTACCAGAAACACAGGTGGTGCATGGTTGTCGTCAGCTCGTGTCGTGAGATGTTAGGTTAAGTCCTAAAACGAGCGAAACCCTTATCGTTAGTTGCCAGCACGTTATGGTGGGGACTTTAACGAGACTGCCAATGATAAATTGGAGGAAGGTGAGGATCACGTCAAATCAGCATGCCCCTTATGACCTGGGCTACAAACGTGATACAATGGCTGTTACAAAGGGTAGCTGAAACGCAAGTTCTTGGCCAATCCCCAAAAACAGTCCCAGTCCGGATTGAAGTCTGCAA

**>AY332658 Peach yellows phytoplasma strain PY-In2 16S ribosomal RNA gene, partial sequence**

AAGAGTTTGATCCTGGCTCAGGATTAACGCTGGCGGCGCGCCTAATACATGCAAGTCGAACGGAGACCCCTCAAAAGGTCTTAGTGGCGAACGGGTGAGTAACACGTAAGTAACCTACCTTTAAGACGAGGATAACAATCGGAAACGGTTGCTAAGACTGGATAGGAAATAAAAAGGCATCTTTTTGTTTTTAAAAGACCTTCTTCGGAGGGTATGCTTAAAGAGGGGCTTGCGCCACATTAGTTAGTTGGTGAGGTAAAGGCTTACCAAGATTATGATGTGTAGTTGGACTGAGAGGTTGAACAGCCACATTGGGACTGAGACACGGCCCAAACTCCTACGGGAGGCAGCAGTAGGGAATTTTCGGCAATGGAGGAAACTCTGACCGAGCGACGCCGCGTGAACGATGAAGTATTTCGGTATGTAAAGTTCTTTTATTGAAGAAGAAAAAATAGTGGAAAAACTATCTTGACGTTATTCAATGAATAAGCCCCGGCTAACTATGTGCCAGCAGCCGCGGTAAGACATAGGGGGCGAGCGTTATCCGGAATTATTGGGCGTAAAGGGTGCGTAGGCGGTTAGATAAGTCTATAATTTAATTTCAGTGCTTAACGCTGTCTTGTTATAGAAACTGTCTTGACTAGAGTGAGATAGAGGCAAGCGGAATTCCATGTGTAGCGGTAAAATGTGTAAATATATGGAGGAACACCAGAAGCGTAGGCGGCTTGCTGGGTCTTTACTGACGCTGAGGCACGAAAGCGTGGGGAGCAAACAGGATTAGATACCCTGGTAGTCCACGCTGTAAACGATGAGTACTAAGTGTCGGGGCAACTCGGTACTGAAGTTAACACATTAAGTACTCCGCCTGAGTAGTACGTACGCAAGTATGAAACTTAAAGGAATTGACGGGACTCCGCACAAGCGGTGGATCATGTTGTTTAATTCGAAGATACACGAAAAACCTTACCAGGTCTTGACATACTCTGCGAAGCTATAGAAATATAGTGGAGGTTATCAGGGATACAGGTGGTGCATGGTTGTCGTCAGTTCGTGTCGTGAGATGTTAGGTTAAGTCCTAAAACGAACGCAACCCCTGTCGTTAGTTACCAGCACGTAATGGTGGGGACTTTAGCGAGACTGCCAATTAAACATTGGAGGAAGGTGGGGATAACGTCAAATCATCATGCCCCTTATGATCTGGGCTACAAACGTGATACAATGGCTGTTACAAAGAGTAGCTGAAACGCGAGTTTTTAGCCAATCTCAAAAAGACAGTCTTAGTCCGGATTGAAGTCTGCAACTCGACTTCATGAAGCTGGAATCGCTAGTAATCGCGAATCAGCATGTCGCGGTGAATACGTTCTCGGGGTTTGTACACACCGCCCGTCAAACCACGAAAGTTAGCAATACCCGAAAGCAGTGGCTTAACTTCGCAAGAAGAGGGAGCTGTCTAAGGTAGGGTTGATGATTGGGGTTAAGTCGTAACAAGGTATCCTTACCGGAAGGTGAGGATGGATCACCTCCTTT

**>AY265214 Chrysanthemum yellows phytoplasma strain CHRYM 16S ribosomal RNA gene, partial sequence**

AAGAGTTTGATCCTGGCTCAGGATTAACGCTGGCGGCGTGCCTAATACATGCAAGTCGAACGGAAGTTTAAGCAATTAAACTTTAGTGGCGAACGGGTGAGTAACGCGTAAGCAATCTGCCCCTAAGACGAGGATAACAGTTGGAAACGACTGCTAAGACTGGATAGCAGACAAGAAGGCATCTTCTTGTTTTTAAAAGACCTATTAATAGGTATGCTTAGGGAGGAGCTTGCGTCACATTAGTTAGTTGGTGGGGTAAAGGCCTACCAAGACTATGATGTGTAGCCGGGCTGAGAGGTTGAACGGCCACATTGGGACTGAGACACGGCCCAAACTCCTACGGGAGGCAGCAGTAGGGAATTTTCGGCAATGGAGGAAACTCTGACCGAGCAACGCCGCGTGAACGATGAAGTATTTCGGTACGTAAAGTTCTTTTATTAGGGAAGAATAAATGATGGAAAAATCATTCTGACGGTACCTAATGAATAAGCCCCGGCTAACTATGTGCCAGCAGCCGCGGTAATACATAGGGGGCAAGCGTTATCCGGAATTATTGGGCGTAAAGGGTGCGTAGGCTGTTAAATAAGTTTATGGTCTAAGTGCAATGCTCAACATTGTGATGCTATAAAAACTGTTTAGCTAGAGTAAGATAGAGGCAAGTGGAATTCCATGTGTAGTGGTAAAATGCGTAAATATATGGAGAGAACACCAGTAGCGAAGCGGCTTGCTGGGTCTTTACTGACGCTGAGGCACGAAAGCGTGGGGAGCAAACAGGATTAGATACCNTGGTAGTCCACGCCGTAAACGATGAGTACTAAACGTTGGGTAAAACCAGTGTTGAAGTTAACACATTAAGTACTCCGCCTGAGTAGTACGTACGCAAGTATGAAACTTAAAGGAATTGACGGGACTCCGCACAAGCGGTGGATCATGTTGTTTAATTCGAAGGTACCCGAAAAACCTCACCAGGTCTTGACATGCTTCTGCAAAGCTGTAGAAACACAGTGGAGGTTATCAGTTGCACAGGTGGTGCATGGTTGTCGTCAGCTCGTGTCGTGAGATGTTGGGTTAAGTCCCGCAACGAGCGCAACCCTTATTGTTAGTTGCCAGCACGTAATGGTGGGGACTTTAGCAAGACTGCCAGTGATAAATTGGAGGAAGGTGGGGACGACGTCAAATCATCATGCCCCTTATGACCTGGGCTACAAACGTGATACAATGGCTGTTACAAAGGGTAGCTGAAACGCAAGTTTTTGGCGAATCTCAAAAAAACAGTCTCAGTTCGGATTGAAGTCTGCAACTCGACTTCATGAAGTTGGAATCGCTAGTAATCGCGAATCAGCATGTCGCGGTGAATACGTTCTCGGGGTTTGTACACACCGCCCGTCAAACCACGAAAGTTGGCAATACCCAAAACCGGTGGCCTAACTTCGCAAGAAGAGGGAACCGTCTAAGGTAGGGTCGATGATTGGGGTTAAGTCGTAACAAGGTATCCCTACCGGAAGGTGGGGATGGATCACCTCCTTT

**>AJ542544 Candidatus Phytoplasma prunorum 16S rRNA gene, tRNA-Ile gene and 23S rRNA gene (partial), strain ESFY-G1**

AGAGTTTGATCCTGGCTCAGGATGAACGCTGGCGGCGTGCTTAATACATGCAAGTCGAACGGAAACTTTTAGTTTCAGTGGCGAACGGGTGAGTAACACGTAAGTAACCTGCCTCTCAGACGAGGATAACAGTTGGAAACGACTGCTAAGACTGGATATGAAGTTTTGAGGCATCTCGAAACTTTTAAAAGACCCGCAAGGGTATGCTGAGAGATGGGCTTGCGGCACATTAGTTAGTTGGTAAGGTAATGGCTTACCAAGACTATGATGTGTAGCTGGACTGAGAGGTTGAACGGCCACATTGGGACTGAGATACGGCCCAAACTCCTACGGGAGGCAGCAGTAAGGAATTTTCGGCAATGGAGGAAACTCTGACCGAGCAACGCCGCGTGAACGATGAAGTATTTAGGTACGTAAAGTTCTTTTATTAAAGAAGAAAAAATGATGGAAAAATCATTCTGACGGTATTTAATGAATAAGCCCCGGCTAACTATGTGCCAGCAGCTGCGGTAATACATGGGGGGCAAGCGTTATCCGGATTTATTGGGCGTAAAGGGTGCGTAGGCGGTTAAATAAGTCTATGGTATAAGTTCAACGCTTAACGTTGTGATGCTATAGAAACTGTTTAACTAGAGTTGGATAGAGGCAAGTGGAATTCCATGTGTAGCGGTAAAATGCGTAAATATATGGAGGAACACCAGTAGCGAAGGCGGCTTGCTGGGTCTTAACTGACGCTGAGGCACGAAAGCGTGGGGAGCAAACAGGATTAGATACCCTGGTAGTCCACGCCGTAAACGATGAGTACTAAGTGTTGGGTTAAACCAGTGCTGAAGTTAACACATTAAGTACTCCGCCTGAGTAGTACGTACGCAAGTATGAAACTTAAAGGAATTGACGGGACTCCGCACAAGCGGTGGATCATGTTGTTTAATTCGAAGATACACGAAAAACCTTACCAGGTCTTGACATACTCTGCAAAGCTATAGAAATATAGTGGAGGTTATCAGGGATACAGGTGGTGCATGGTTGTCGTCAGCTCGTGTCGTGAGATGTTGGGTTAAGTCCCGCAACGAGCGCAACCCTTATCACTAGTTACCATCATTTAGTTGGGCACTTTAGTGAGACTGCCAATGATAAATTGGAGGAAGGTGGGGATTACGTCAAATCATCATGCCCCTTATGACCTGGGCTACAAACGTGATACAATGGCTGTTACAAAGAGTAGCTGAAACGTGAGTTTTTAGCAAATCTCAAAAAAACAGTCTCAGTTCGGATTGAAGTCTGCAACTCGACTTCATGAAGTCGGAATCGCTAGTAATCGCGAATCAGCATGTCGTGGTGAATACGTTCTCGGGGTTTGTACACACCGCCCGTCAAACCACGAAAGTTGACAATACCCAAAACCAGTAGCCTAACTTGCAAAAGAGGGAACTGTCTAAGGTAGGGTTGATGATTGGGGTTAAGTCGTAACAAGGTATCCCTACCGGAAGGTGGGGATGGATCACCTCCTTTCTAAGGAAAATATCATCTTCAGTTGTGAAAGACTTAAAAAAAGTTTTTTATTTTTTAAGATAAAAATCAATAATGGCTTGGGCCTATAGCTCAGTTGGTTAGAGCACACGCCTGATAAGCGTGAGGTCGATGGTTCGAGTCCATTTAGGCCCACCAAAATATTTATTTTAAAAAAACAAGCTCTTTGAAAAGTAGATAAATTAAGGTTAGAAGAATTAAAGAAATTAAGGGCGCACAGTGGATGCCTTGGCACTAAGAGCCGATGAAGGACG

**>AF322644 Aster yellows phytoplasma strain AY1 clone 14 ribosomal RNA operon A, partial sequence**

AAGAGTTTGATCCTGGCTCAGGATTAACGCTGGCGGCGTGCCTAATACATGCAAGTCGAACGGAAGTTTAAGCAATTAAACTTTAGTGGCGAACGGGTGAGTAACGCGTAAGCAATCTGCCCCTAAGACGAGGATAACAGTTGGAAACGACTGCTAAGACTGGATAGGAGACAAGAGGGCATCTTCTTGTTTTTAAAAGACCTAGCAATAGGTATGCTTAGGGAGGAGCTTGCGTCACATTAGTTAGTTGGTGGGGTAAAGGCCTAACAAGACTATGATGTGTAGCCGGGCTGAGAGGTTGAACGGCCACATTGGGACTGAGACACGGCCCAAACTCCTACGGGAGGCAGCAGTAGGGAATTTTCGGCAATGGAGGAAACTCTGACCGAGCAACGCCGCGTGAACGATGAAGTATTTCGGTACGTAAAGTTCTTTTATTAGGGAAGAATAAATGATGGAAAAATCATTCTGACGGTACCTAATGAATAAGCCCCGGCTAACTATGTGCCAGCAGCCGCGGTAATACATAGGGGGCAAGCGTTATCCGGAATTATTGGGCGTAAAGGGTGCGTAGGCGGTTAAATAAGTTTATGGTCTAAGTGCAATGCTCAACATTGTGATGCTATAAAAACTGTTTAGCTAGAGTAAGATAGAGGCAAGTGGAATTCCATGTGTAGTGGTAAAATGCGTAAATATATGGAGGAACACCAGTAGCGAAGGCGGCTTGCTGGGTCTTTACTGACGCTGAGGCACGAAAGCGTGGGGAGCAAACAGGATTAGATACCCTGGTAGTCCACGCCGTAAACGATGAGTACTAAACGTTGGGTAAAACCAGTGTTGAAGTTAACACATTAAGTACTCCGCCTGAGTAGTACGTACGCAAGTATGAAACTTAAAGGAATTGACGGGACTCCGCACAAGCGGTGGATCATGTTGTTTAATTCGAAGGTACCCGAAAAACCTCACCAGGTCTTGACATGCTTCTGCAAAGCTGTAGAAACACAGTGGAGGTTATCAGTTGCACAGGTGGTGCATGGTTGTCGTCAGCTCGTGTCGTGAGATGTTGGGTTAAGTCCCGCAACGAGCGCAACCCTTATTGTTAGTTACCAGCACGTAATGGTGGGGACTTTAGCAAGACTGCCAGTGATAAATTGGAGGAAGGTGGGGACGACGTCAAATCATCATGCCCCTTATGACCTGGGCTACAAACGTGATACAATGGCTGTTACAAAGGGTAGCTGAAGCGCAAGTTTTTGGCGAATCTCAAAAAAACAGTCTCAGTTCGGATTGAAGTCTGCAACTCGACTTCATGAAGTTGGAATCGCTAGTAATCGCGAATCAGCATGTCGCGGTGAATACGTTCTCGGGGTTTGTACAAACCGCCCGTCAAACCACGAAAGTTGGTAATACCCAAAGCCGGTGGCCTAACTTCGCAAGAAGAGGGAACCGTCTAAGGTAGGGTCGATGATTGGGGTTAAGTCGTAACAAGGTATCCCTACCGGAAGGTGGGGATGGATCACCTCCTTTCTAAGGAAACAATTATCATCTTCAGTTTTGAGAGACTTAAGAAAGTTTTTCATTGTAACTTGCTTGCAAATTGTATTTGCAACATTTTAATCTTTTTAAGATTAAGGGCCTATAGCTCAGTTGGTTAGAGCACACGCCTGATAAGCGTGAGGTCGGTGGTTCAAGTCCATTTAGGCCCACCATAACCACAAATAGGCAAAATCTTAAAAAAGCTCTTTGAAAAGTAGATAAACGAAGGTTAAAAAATCAAAGGAACTAAGGGCGCACAGTGGATGCCTTGGCACTAAGAGCCGATGAAGGACG

**>AF222064 Tomato big bud phytoplasma ribosomal RNA operon, partial sequence**

AAGAGTTTGATCCTGGCTCAGGATTAACGCTGGCGGCGTGCCTAATACATGCAAGTCGAACGGAAGTTTAATCAATTAAACTTTAGTGGCGAACGGGTGAGTAACGCGTAAGCAATCTACCCCTAAGACGAGGATAACAGTTGGAAACGACTGCTAAGACTGGATAGTAGACAAGAAGGCATCTTCTTGTTTTTAAAAGACCTATTAATAGGTATGCTTAGGGAGGAGCTTGCGTCACATTAGTTAGTTGGTGGGGTAAAGGCCTACCAAGACTATGATGTGTAGCCGGGCTGAGGGGTTGAACGGCCACATTGGGACTGAGACACGGCCCAAACTCCTACGGGAGGCAGCAGTAGGGAATTTTCGGCAATGGAGGAAACTCTGACCGAGCAACGCCGCGTGAACGATGAAGTATTTCGGTACGTAAAGTTCTTTTATTAGGGAAGAATAAATGATGGAAAAATCATTCTGACGGTACCTAATGAATAAGCCCCGGCTAACTATGTGCCAGCAGCCGCGGTAATACATAGGGGGCAAGCGTTATCCGGAATTATTGGGCGTAAAGGGTGCGTAGGCTGTTAAATAAGTTTATGGTCTAAGTGCAATGCTCAACATTGTGATGCTATAAAAACTGTTTAGCTAGAGTAAGATAGAGGCAAGTGGAATTCCATGTGTAGTGGTAAAATGCGTAAATATATGGAGGAACACCAGTAGCGAAGGCGGCTTGCTGGGTCTTTACTGACGCTGAGGCACGAAAGCGTGGGGAGCAAACAGGATTAGATACCCTGGTAGTCCACGCCGTAAACGATGAGTACTAAACGTTGGGTAAAACCAGTGTTGAAGTTAACACATTAAGTACTCCGCCTGAGTAGTACGTACGCAAGTATGAAACTTAAAGGAATTGACGGGACTCCGCACAAGCGGTGGATCATGTTGTTTAATTCGAAGGTACCCGAAAAACCTCACCAGGTCTTGACATGCTTCTGCAAAGCTGTAGAAACACAGTGGAGGTTATCAGTTGCACAGGTGGTGCATGGTTGTCGTCAGCTCGTGTCGTGAGATGTTGGGTTAAGTCCCGCAACGAGCGCAACCCTTATTGTTAGTTGCCAGCACGTAATGGTGGGGACTTTAGCAAGACTGCCAGTGATAAATTGGAGGAAGGTGGGGACGACGTCAAATCATCATGCCCCTTATGACCTGGGCTACAAACGTGATACAATGGCTGTTACAAAGGGTAGCTGAAACGCAAGTTTTTGGCGAATCTCAAAAAAACAGTCTCAGTTCGGATTGAAGTCTGCAACTCGACTTCATGAAGTTGGAATCGCTAGTAATCGCGAATCAGCATGTCGCGGTGAATACGTTCTCGGGGTTTGTACACACCGCCCGTCAAACCACGAAAGTTGGCAATACCCAAAACCGGTGGCCTAACTTCGCAAGAAGAGGGAACCGTCTAAGGTAGGGTCGATGATTGGGGTTAAGTCGTAACAAGGTATCCCTACCGGAAGGTGGGGATGGATCACCTCCTTTCTAAGGAAACAATTATCATCTTCAGTTTTGAGAGACTTAAGAAAGTTTTTCATTTTAAATTCCTTGCAAATTTTATTTGCAACATTTAAATCTTTTTAAGATTAAGGGCCTATAGCTCAGTTGGTTAGAGCACACGCCTGATAAGTGTGAGGTCGGTGGTTCAAGTCCATTTAGGCCCACCATAACCAAAAATAGGCAAAATCTTAAAAAAGCTCTTTGAAAAGTAGATAAACGAAGGTTAAAAAATCAAAGGAACTAAGGGCGCACAGCGGATGCCTTGGCACTAAGAGCCGATGAAGGACG

**>AF147708 Hibiscus witches'-broom phytoplasma strain HibWB26 16S ribosomal RNA gene, 16S-23S ribosomal RNA intergenic spacer region, and tRNA-Ile gene, complete sequence; 23S ribosomal RNA gene, partial sequence**

AAGAGTTTGATCCTGGCTCAGGATTAACGCTGGCGGCGTGCCTAATACATGCAAGTCGAACGGAAACTTTCGGGTTTTAGTGGCGAACGGGTGAGTAACACGTAAGCAACCTGCCCTAATGACGAGGATAACCATTGGAAACAATGGCTAAGACTGGATAGGAAAAAGAAAGGCATCTTTCTTTTTTTAAAAGACCTAGTAATAGGTATGCGTTAGGAGGGGCTTGCGCCATATTAGTTAGTTGGTAAGGTAACGGCTTACCAAGACGATGATGTGTAGCTGGACTGAGAGGTTGAACAGCCACATTGGGACTGAGACACGGCCCAAACTCCTACGGGAGGCAGCAGTAAGGAATTTTCGGCAATGGAGGCAACTCTGACCGAGCAACGCCGCGTGAACGACGAAGTACTTCGGTATGTAAAGTTCTTTTATCAAAGAAGAAAAGCAAATGGCGAACCATTTGTCTGCCGGTATTTGATGAATAAGCCCCGGCTAATTATGTGCCAGCAGCCGCGGTAAGACATAAGGGGCAAGTGTTATCCGGAATTATTGGGCGTAAAGGGTGCGTAGGCGGCAGAGTAAGTCAGTGGTGTAATGGCAACGCTTAACGTTGTCAGGCTATTGAAACTACTTTGCTAGAGTTGGATAGAGGCGAGTGGAATTCCATGTGTAGCGGTAAAATGCGTAAATATATGGAGGAACACCAGAGGCGTAGGCGGCTCGCTGGGTCTTAACTGACGCTGAGGCACGAAAGCGTGGGGAGCAAACAGGATTAGATACCCTGGTAGTCCACGCCGTAAACGATGAGTACTAAGTGTCGGGTCAAAACCGGTACTGAAGTTAACACATTAAGTACTCCGCCTGAGTAGTACGTACGCAAGTATGAAACTTAAAGGAATTGACGGGACTCCGCACAAGCGGTGGATCATGTTGTTTAATTCGAAGATACCCGAAAAACCTTACCAGGTCTTGACATGTTCTTGCGAAACGGTAGTAATATCGTGGAGGTTATCAGGAACACAGGTGGTGCATGGTTGTCGTCAGCTCGTGTCGTGAGATGTTAGGTTAAGTCCTAAAACGAGCGAAACCCTTATCGTTAGTTGCCAGCACGTTATGGTGGGGACTTTAACGAGACTGCCAATGATAAATTGGAGGAAGGTGAGGATCACGTCAAATCAGCATGCCCCTTATGACCTGGGCTACAAACGTGATACAATGGCTGCTACAAAGGGTAGCTGAAACGCAAGTTCTTGGCCAATCCCCAAAAGCAGTCTCAGTCCGGATTGAAGTCTGCAACTCGACTTCATGAAGTTGGAATCGCTAGTAATCGCGAATCAGCATGTCGCGGTGAATACGTTCTCGGGGTTTGTACACACCGCCCGTCAAACCACGAAAGTTGGCAATACCCCAAAACGGTAGCCTAACTCGGTTTTCCGAGAGGGCGCCGTCTAAGGTAGGGTTGATGATTGGGGTTAAGTCGTAACAAGGTATCCCTACCCGGAAGGTGGGGATGGATCACCTCCTTTCTAAGGAATTAAGATAAAATCAATCATCTTCAGTTTTGAAAGACTTAGAAATTAAGTCTTTCTTCGTGACTTTACGAAGGGCCTATAGCTCAGTCGGTTAGAGCACACGCCTGATAAGCGTGAGGTCGGTGGTTCAAATCCATTTAGGCCCACCAGAGTTATAGGTTTCGTTAAAAACACAAATTCTTTGAAAATTAGGTAAATAAATAGTTATTTATCGGAACATTAAAGGAAATAAGAGCGCACAGTGGATGCCTAGGCACCAAGAGCCGATGAAGGACG

**>AF092209 Ash yellows phytoplasma 16S ribosomal RNA gene, 16S-23S ribosomal RNA intergenic spacer region, and tRNA-Ile gene, complete sequence**

GCGTGCCTAATACATGCAAGTCGAACGGAAACCCCTCAAAAGGTTTTAGTGGCGAACGGGTGAGTAACACGTAAGTAATCTACCTTTAAGACGAGGATAACAACCGGAAACAGTTGCTAAGACTGGATAGGAAATAAAAAGGCATCTTTTTATTTTTAAAAGACCTTTTTCGAAAGGTATGCTTAAAGATGAGCTTGCGCCACATTAGTTTGTTGGTGGGGTAATGGCCTACCAAGACGATGATGTGTAGCTGGACTGAGAGGTCGAACAGCCACATTGGGACTGAGACACGGCCCAAACTCCTACGGGAGGCAGCAGTAGGGAATTTTCGGCAATGGAGGAAACTCTGACCGAGCAACGCCGCGTGAACGATGAAGTATTTCGGTATGTAAAGTTCTTTTATTGAAGAAGAAAAAATAGTGGAAAAACTATCTTGACGTTATTCAATGAATAAGCCCCGGCTAACTATGTGCCAGCAGCCGCGGTAAGACATAGGGGGCGAGCGTTATCCGGAATTATTGGGCGTAAAGGGTGCGTAGGCGGTTAGGAAAGTCTATAATTTAATTTCAGTGCTTAACGCTGTCTTGTTATAGAAACTACCTTGACTAGAGTTAGATAGAGGCAAGCGGAATTCCATGTGTAGCGGTAAAATGTGTAAATATATGGAGGAACACCAGAAGCGTAGGCGGCTTGCTGGGTCTTGACTGACGCTGAGGCACGAAAGCGTGGGTAGCAAACAGGATTAGATACCCTGGTAGTCCACGCCGTAAACGATGAGTACTAAGTGTCGGGATAAAACTCGGTACTGAAGTTAACACATTAAGTACTCCGCCTGAGTAGTACGTACGCAAGTATGAAACTTAAAGGAATTGACGGGACTCCGCACAAGCGGTGGATCATGTTGTTTAATTCGAAGATACACGAAAAATCTTACCAGGTCTTGACATGCTCTGCAAAGCTATAGAAATATAGTGGAGGTTATCAGGGACACAGGTGGTGCATGGTTGTCGTCAGTTCGTGTCGTGAGATGTTAGGTTAAGTCCTAAAACGAGCGCAACCCTTGTCGTTAGTTACCAGCACGTAATGGTGGGCACTTTAACGAGACTGCCAATTAAACATTGGAGGAAGGTGAGGATTACGTCAAATCATCATGCCCCTTATGATCTGGGCTACAAACGTGATACAATGGCTGTTACAAAGAGTAGCTGAAGCGCGAGTTTTTAGCCAATCTCAAAAAAGCAGTCTCAGTTCGGATTGAAGTCTGTAACTCGACTTCATGAAGTTGGAATCGCTAGTAATCGCGAATCAGCATGTCGCGGTGAATACGTTCTCGGGGTTTGTACACACCGCCCGTCAAACCACGAAAGTTAGCAATACCCGAAAGCAGCAGCTTAACTTCGCAAGAAGAGGGAACTGTCTAAGGTAGGGTTGATGATTGGGGTTAAGTCGTAACAAGGTATCCCTACCGGAAGGTGGGGATGGATCACCTCCTTTCTAAGGATAATACATAAATTCATCATCTTCAGTTTTGAAAGACTTAGATTGAAAAAATAAGTTTTTCTTTTTTAAGCACCAAAGGGCCTATAGCTCAGTTGGTTAGAGCACACGCCTGATAAGCGTGAGGTCGGTGGTTCAAGTCCATTTAGGCCCACCAATAGATTAATATAAACGGTCCTGCTTAAAAGAAAGTTCTTTGAAAAGTAGATAAATATAGCTTTTAAA

**>AB819337 Candidatus Phytoplasma oryzae gene for 16S ribosomal RNA, partial sequence, isolate: Takamatsu RYD41**

TCAGGATTAACGCTGGCGGCGTGCTTAATACATGCAAGTCGAACGGAAATTTTCGGATTTTAGTGGCGAACGGGTGAGTAACACGTAAGTAACCTGCCTTTAAGACGAGGATAACAATTGGAAACAGTTGCTAAAACTGGATAGGAAATTAAAAGGTATCTTTTAATTTTTAAAAGACCTTCTTTCGAAGAGTATACTTAAAGAGGGGCTTGCGGCACATTAGTTAGTTGGTAGGGTAATGGCCTACCAAGACTATGATGTGTAGCTGGACTGAGAGGTTGAACAGCCACATTGGGACTGAGACACGGCCCAAACTCCTACGGGAGGCAGCAGTAGGGAATTTTCGGCAATGGAGGAAACTCTGACCGAGCAACGCCGCGTGAACGATGAAGTATTTCGGTATGTAAAGTTCTTTTATTGAAGAAGAAAAAATAGTGGAAAAACTATCTTGACGCTATTCAATGAATAAGCCCCGGCAAACTATGTGCCAGCAGCCGCGGTAATACATAGGGGGCAAGCGTTATCCGGAATTATTGGGCGTAAAGGGTGCGTAGGCGGTTTGATAAGTCTATAGTTTAATTTCAGTGCTTAACACTGTCCTGCTATAGAAACTATCAGACTAGAGTGAGATAGAGGCAAGTGGAATTCCATGTGTAGCGGTAAAATGCGTAAATATATGGAGGAACACCAGAGGCGTAGGCGGCTTGCTGGGTCTTTACTGACGCTGAGGCACGAAAGCGTGGGGAGCAAACAGGATTAGATACCCTGGTAGTCCACGCCGTAAACGATGAGTACTAAGTGTCGGGGGAACTCGGTACTGAAGTTAACACATTAAGTACTCCGCCTGAGTAGTACGTACGCAAGTATGAAACTTAAAGGAATTGACGGGACTCCGCACAAGCGGTGGATCATGTTGTTTAATTCGAAGATACACGAAAAACCTTACCAGGTCTTGACATACTCTGCAAAGCTATAGCAATATAGTGGAGGTTATCAGGGATACAGGTGGTGCATGGTTGTCGTCAGCTCGTGTCGTGAGATGTTAGGTTAAGTCCTAAAACGAGCGCAACCCTTGTCATTAGTTGCCAGCATGTTATGATGGGCACTTTAATGAGACTGCCAATAAAAAATTGGAGGAAGGTGAGGATCACGTCAAGTCATCATGCCCCTTATGATCTGGGCTACAAACGTGATACAATGGCTGTTACAAAGAGTAGCTAAAACGTAAGTTCAAGCCAATCTCAAAAAAACAGTCTCAGTTCGGATTGAAGTCTGCAACTCGACTTCATGAAGTTGGAATCGCTAGTAATCGCGAATCAGCATGTCGCGGTGAATACGTTCTCGG

**>AB558132 Sesame phyllody phytoplasma genes for 16S rRNA, 16S-23S rRNA intergenic spacer, 23S rRNA, partial and complete sequence**

AGAGTTTGATCCTGGCTCAGGATTAACGCTGGCGGCGTGCCTAATACATGCAAGTCGAACGGAAGTTTAAGCAATTAAACTTTAGTGGCGAACGGGTGAGTAACGCGTAAGCAATCTGCCCCTAAGACGAGGATAACAGTTGGAAACGACTGCTAAGACTGGATAGGAGACAAGAGGGCATCTTCTTGTTTTTAAAAGACCTAGCAATAGGTATGCTTAGGGAGGAGCTTGCGTCACATTAGTTAGTTGGTGGGGTAAAGGCCTACCAAGACTATGATGTGTAGCCGGGCTGAGAGGTTGAACGGCCACATTGGGACTGAGACACGGCCCAAACTCCTACGGGAGGCAGCAGTAGGGAATTTTCGGCAATGGAGGAAACTCTGACCGAGCAACGCCGCGTGAACGATGAAGTATTTCGGTACGTAAAGTTCTTTTATTAGGGAAGAATAAATGATGGAAAAATCATTCTGACGGTACCTAATGAATAAGCCCCGGCTAACTATGTGCCAGCAGCCGCGGTAATACATAGGGGGCAAGCGTTATCCGGAATTATTGGGCGTAAAGGGTGCGTAGGCGGTTAAATAAGTTTATGGTCTAAGTGCAATGCTCAACATTGTGATGCTATAAAAACTGTTTAGCTAGAGTAAGATAGAGGCAAGTGGAATTCCATGTGTAGTGGTAAAATGCGTAAATATATGGAGGAACACCAGTAGCGAAGGCGGCTTGCTGGGTCTTTACTGACGCTGAGGCACGAAAGCGTGGGGAGCAAACAGGATTAGATACCCTGGTAGTCCACGCCGTAAACGATGAGTACTAAACGTTGGGTAAAACCAGTGTTGAAGTTAACACATTAAGTACTCCGCCTGAGTAGTACGTACGCAAGTATGAAACTTAAAGGAATTGACGGGACTCCGCACAAGCGGTGGATCATGTTGTTTAATTCGAAGGTACCCGAAAAACCTCACCAGGTCTTGACATGCTTCTGCAAAGCTGTAGAAACACAGTGGAGGTTATCAGTTGCACAGGTGGTGCATGGTTGTCGTCAGCTCGTGTCGTGAGATGTTGGGTTAAGTCCCGCAACGAGCGCAACCCTTATTGTTAGTTACCAGCACGTAATGGTGGGGACTTTAGCAAGACTGCCAGTGATAAATTGGAGGAAGGTGGGGACGACGTCAAATCATCATGCCCCTTATGACCTGGGCTACAAACGTGATACAATGGCTGTTACAAAGGGTAGCTGAAGCGCAAGTTTTTGGCGAATCTCAAAAAAACAGTCTCAGTTCGGATTGAAGTCTGCAACTCGACTTCATGAAGTTGGAATCGCTAGTAATCGCGAATCAGCATGTCGCGGTGAATACGTTCTCGGGGTTTGTACACACCGCCCGTCAAACCACGAAAGTTGGYAATACCCAAAGCCGGTGGCCTAACTTCGCAAGAAGAGGGAACCGTCTAAGGTAGGGTCGATGATTGGGGTTAAGTCGTAACAAGGTATCCCTACCGGAAGGTGGGGATGGATCACCTCCTTTCTAAGGAAACAATTATCATCTTCAGTTTTGAGAGACTTAAGAAAGTTTTTCATTGTAACTTGCTTGCAAATTGTATTTGCAACATTTTAATCTTTTTAAGATTAAGGGCCTATAGCTCAGTTGGTTAGAGCACACGCCTGATAAGCGTGAGGTCGGTGGTTCAAGTCCATTTAGGCCCACCATAACCACAAATAGGCAAAATCTTAAAAAAGCTCTTTGAAAAGTAGATAAACGAAGGTTAAAAAATCAAAGGAACTAAGGGCGCACAGTGGATGCCTTGGCACTAAGAGCCGATGAAGGACGCAATTAACGGCGA
