# Supplementary material for: Amplification-free detection of plant pathogens by improved CRISPR-Cas12a systems: a case study on phytoplasma
Source: Front Plant Sci. 2025 Mar 6;16:1544513. doi: 10.3389/fpls.2025.1544513 (PMC11924941; doi:10.3389/fpls.2025.1544513)
Supplement: Supplementary file 2 [file Presentation1.pptx]

## Slide 1
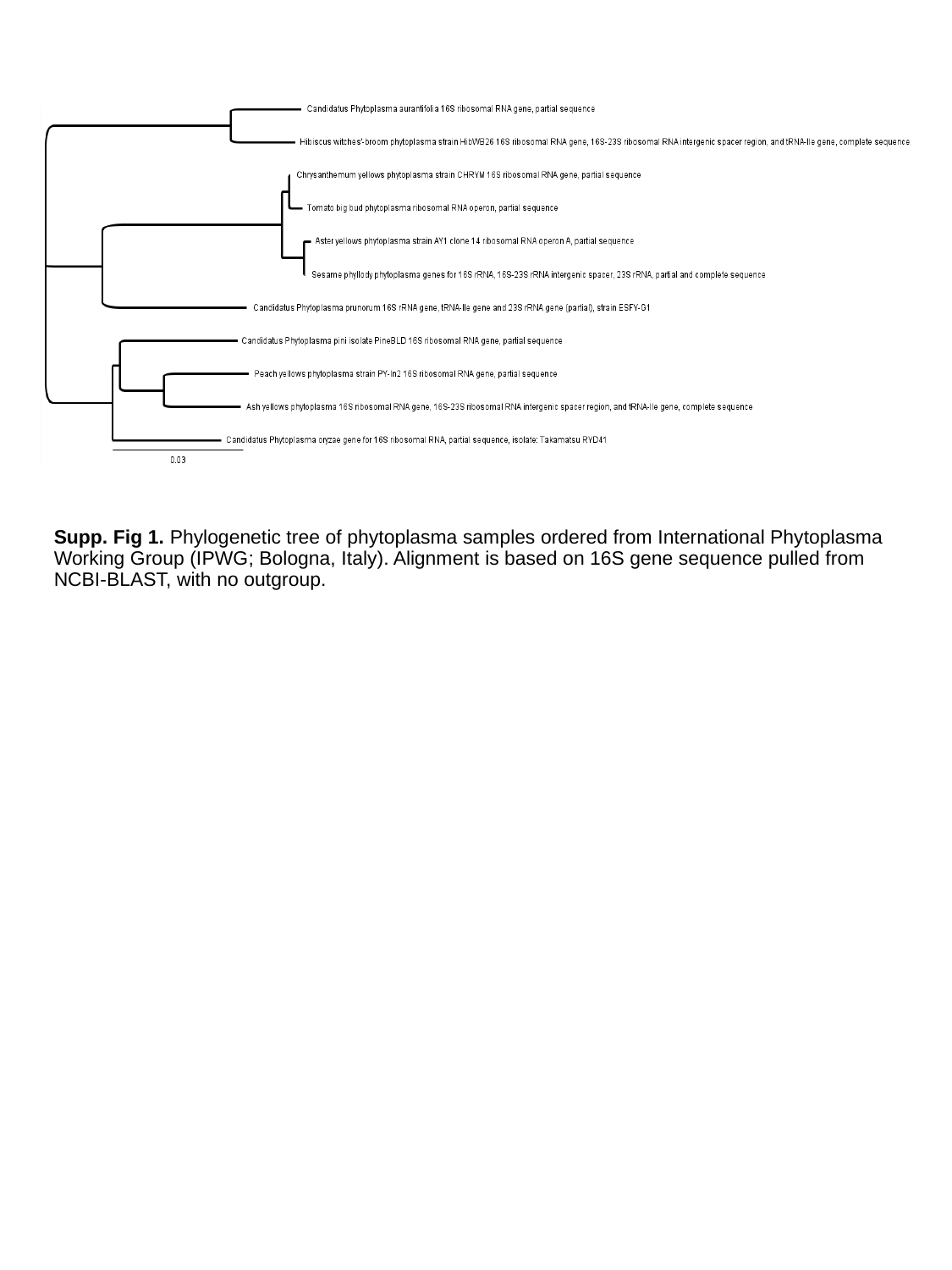

# Supp. Fig 1. Phylogenetic tree of phytoplasma samples ordered from International Phytoplasma Working Group (IPWG; Bologna, Italy). Alignment is based on 16S gene sequence pulled from NCBI-BLAST, with no outgroup.

## Slide 2
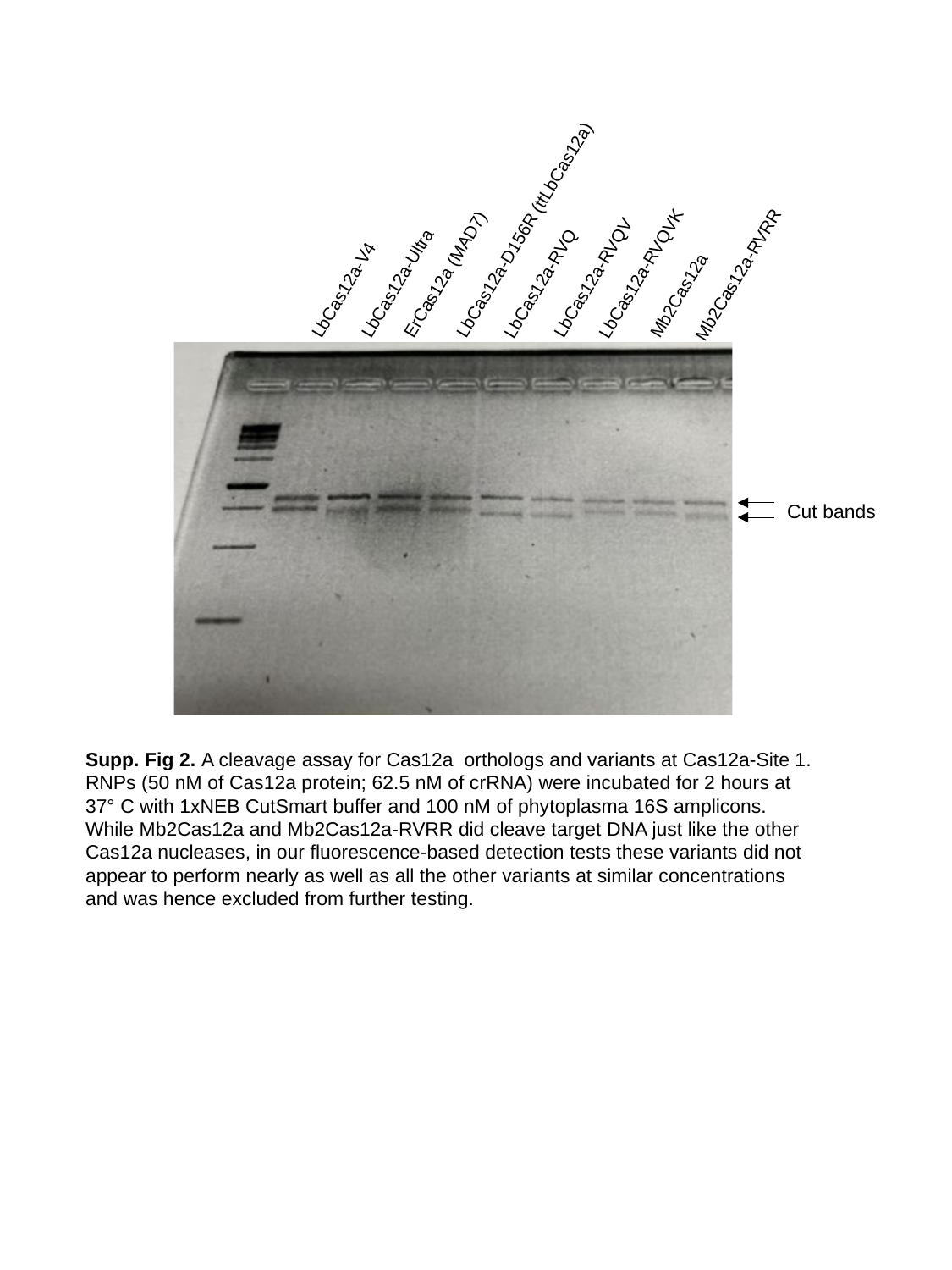

LbCas12a-D156R (ttLbCas12a)
LbCas12a-RVQVK
ErCas12a (MAD7)
Mb2Cas12a-RVRR
LbCas12a-RVQV
LbCas12a-Ultra
LbCas12a-RVQ
LbCas12a-V4
Mb2Cas12a
Cut bands
Supp. Fig 2. A cleavage assay for Cas12a orthologs and variants at Cas12a-Site 1. RNPs (50 nM of Cas12a protein; 62.5 nM of crRNA) were incubated for 2 hours at 37° C with 1xNEB CutSmart buffer and 100 nM of phytoplasma 16S amplicons. While Mb2Cas12a and Mb2Cas12a-RVRR did cleave target DNA just like the other Cas12a nucleases, in our fluorescence-based detection tests these variants did not appear to perform nearly as well as all the other variants at similar concentrations and was hence excluded from further testing.

## Slide 3
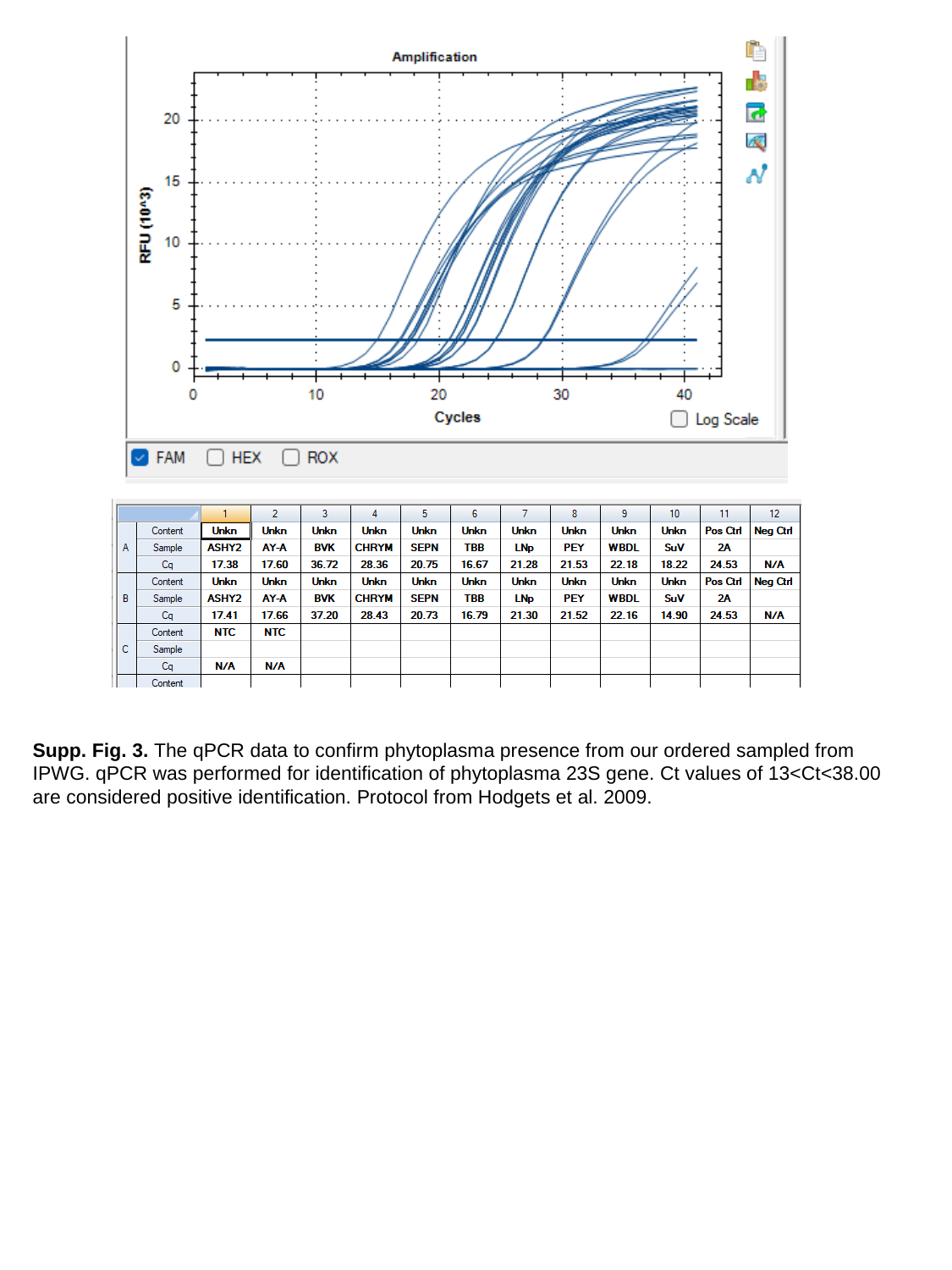

Supp. Fig. 3. The qPCR data to confirm phytoplasma presence from our ordered sampled from IPWG. qPCR was performed for identification of phytoplasma 23S gene. Ct values of 13<Ct<38.00 are considered positive identification. Protocol from Hodgets et al. 2009.

## Slide 4
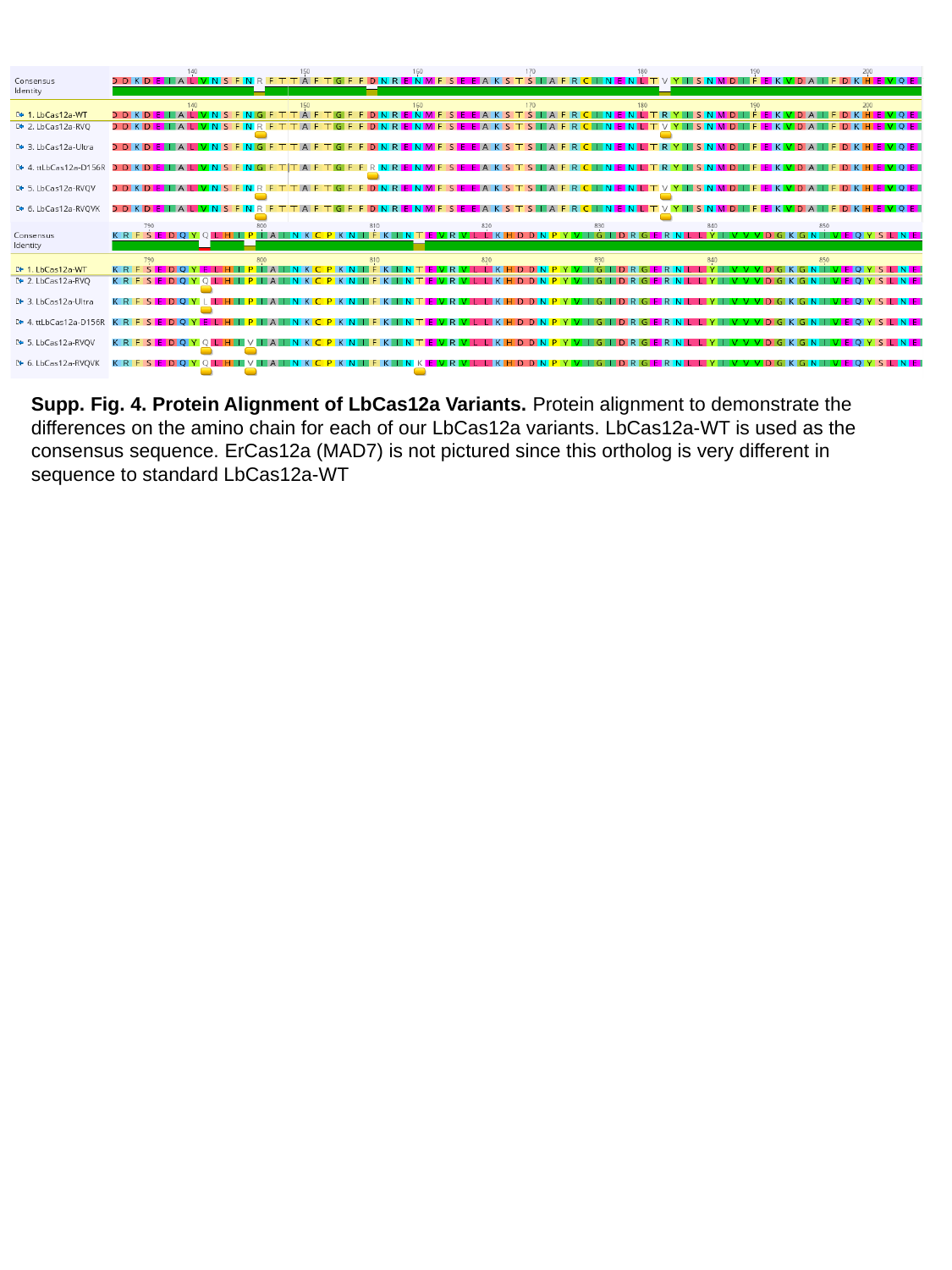

#
Supp. Fig. 4. Protein Alignment of LbCas12a Variants. Protein alignment to demonstrate the differences on the amino chain for each of our LbCas12a variants. LbCas12a-WT is used as the consensus sequence. ErCas12a (MAD7) is not pictured since this ortholog is very different in sequence to standard LbCas12a-WT
